# BHLHE40 Cooperates with GATA2/3 to Control Human Syncytiotrophoblast Lineage Differentiation

**DOI:** 10.1002/advs.202507642

**Published:** 2025-09-05

**Authors:** Lijin Peng, Weijie Zhao, Chunfang Xu, Yue Li, Jiani Guo, Taotao Zhou, Philip Chiu, Huimei Wu, Qingyu Wu, Yanxing Wei, Shaorong Gao, Meirong Du

**Affiliations:** ^1^ Laboratory of Reproduction Immunology Obstetrics and Gynecology Hospital Fudan University Shanghai Medical College Shanghai 200032 China; ^2^ Longgang District Maternity & Child Healthcare Hospital of Shenzhen City (Affiliated Shenzhen Women and Children's Hospital (Longgang) of Shantou University Medical College) Shenzhen Guangdong 518100 China; ^3^ Department of Obstetrics and Gynecology LKS Faculty of Medicine The University of Hong Kong Hong Kong 999077 China; ^4^ Guangxi Reproductive Medicine Institute The First Affiliated Hospital Guangxi Medical University Nanning Guangxi 510120 China; ^5^ Cyrus Tang Hematology Center State Key Laboratory of Radiation Medicine and Prevention Soochow University Suzhou 215123 China; ^6^ Department of Obstetrics and Gynecology Nanfang Hospital Southern Medical University Guangzhou 510515 China; ^7^ Key Laboratory of Functional Proteomics of Guangdong Province School of Basic Medical Sciences Southern Medical University Guangzhou 510515 China; ^8^ Research Centre for Women's and Infants' Health Lunenfeld‐Tanenbaum Research Institute Sinai Health System Toronto M5T3H7 Canada; ^9^ Shanghai Key Laboratory of Maternal Fetal Medicine Shanghai Institute of Maternal‐Fetal Medicine and Gynecologic Oncology Shanghai First Maternity and Infant Hospital School of Medicine Tongji University Shanghai 201204 China; ^10^ School of Life Sciences and Technology Tongji University Shanghai 200092 China

**Keywords:** BHLHE40, GATA2/3, human trophoblast stem cells, syncytiotrophoblasts

## Abstract

Syncytiotrophoblasts (STBs) constitute one of the core components of the placenta, responsible for synthesizing pregnancy‐sustaining hormones such as human chorionic gonadotropin (HCG). Deficient syncytialization of cytotrophoblasts affects the hormonal secretion and placental development, contributing to pregnancy‐associated disorders, including spontaneous miscarriage. To date, the molecular mechanisms, particularly the role of transcription factors (TFs), in STB lineage specification remain incompletely understood. Through targeting direct regulators of a STB lineage‐specific marker, *CGB* (encoding chorionic gonadotropin‐β), by DNA pull‐down coupled with mass spectrometry, basic helix‐loop‐helix family member 40 (BHLHE40) has been identified as a key regulator in human STB differentiation. BHLHE40 expression is increased during STB differentiation but reduced in villous samples from women with miscarriages. CRISPR/Cas9‐mediated knockout of BHLHE40 in human trophoblast stem cells (TSCs) prevents STB differentiation in vitro, impairing gene expression critical for hormone synthesis and cell syncytialization. Mechanistically, BHLHE40 interacts directly with GATA2 and GATA3 to facilitate their chromatin occupancy, thereby activating transcriptional programs essential for STB differentiation. These findings uncover a BHLHE40‐GATA2/3 regulatory network governing human trophoblast lineage commitment, providing insights into placental development and potential therapeutic targets for pregnancy disorders.

## Graphical abstract

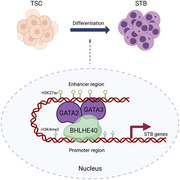

## 1 Introduction

The placenta is a critical connection between the mother and the fetus. Defects in placental development can cause severe gestational disorders, including miscarriage, preeclampsia, preterm birth, and fetal growth restriction, with lifelong consequences for the offspring's health.^[^ ^1^ ^]^ Trophoblasts are a major cell type in the placenta, which undergo dynamic differentiation to gain specific structural and functional features during pregnancy. In early pregnancy, the placental development depends on active proliferation of trophoblast progenitor cells, known as cytotrophoblast (CTB) cells, which diverge into two terminal lineages: invasive extravillous trophoblasts (EVTs) and multinucleated syncytiotrophoblasts (STBs).^[^ ^2^ ^]^ The process to form multinucleated STBs is defined as syncytialization, which is maintained by the constant fusion of CTBs.^[^ ^3^ ^]^ One of the hallmark functions of these terminally differentiated STBs is the production of human chorionic gonadotropin (HCG),^[^ ^2^ ^]^ a pregnancy‐sustaining hormone and a clinical indicator for placental function and fetal development. Insufficient HCG production is an important factor in early pregnancy losses.^[^ ^4^ ^]^ HCG is a heterodimeric protein consisting of an α subunit and a β subunit.^[^ ^5^ ^]^ The α subunit is shared by other hormones (e.g., luteinizing hormones, follicle‐stimulating hormones, and thyroid‐stimulating hormone), whereas the β subunit is HCG‐specific.^[^ ^6^ ^]^ Beta‐HCG is encoded by a set of homologous genes, i.e., *CGB1‐3/5/7/8*, which are often used as a marker for STB identification. Given the pivotal role of STBs in pregnancy, it is necessary to explore the underlying mechanism regulating the differentiation process and hormone synthesis.

One of the major challenges hampering trophoblast differentiation studies is the lack of an ideal in vitro model.^[^ ^7^ ^]^ Recently, trophoblast stem cells (TSCs), derived from human placental trophoblastic tissue^[^ ^8^ ^]^ or pluripotent stem cells,^[^ ^9^ ^]^ have been reported as an optimal model. These cells exhibit sustained proliferative capacity and bidirectional differentiation potential toward STBs and EVTs, enabling mechanistic studies of placental development.^[^ ^10^, ^11^, ^12^, ^13^, ^14^ ^]^ Cell fate transitions are orchestrated by transcription factors (TFs)^[^ ^15^ ^]^ operating within accessible chromatin landscapes. Exiting from the CTB progenitor state involves coordinated suppression of stemness‐maintaining TFs, including TEAD4,^[^ ^16^ ^]^ MSX2,^[^ ^12^, ^17^ ^]^ YAP,^[^ ^18^ ^]^ and TP63,^[^ ^19^ ^]^ coupled with activation of STB differentiation drivers such as GCM1,^[^ ^20^ ^]^ OVOL1,^[^ ^21^ ^]^ TFEB,^[^ ^13^, ^22^ ^]^ and TBX3.^[^ ^10^ ^]^ Moreover, TFAP2A, TFAP2C, GATA2, and GATA3, collectively referred to as “trophectoderm four”, are crucial in both suppression of pluripotency networks and induction of STB lineage‐specific genes.^[^ ^23^, ^24^ ^]^ Studies have indicated functional redundancy between GATA2 and GATA3 in placental development, as the dual knockout mouse models led to early pregnancy failure.^[^ ^25^ ^]^ GATA2 and GATA3 are pivotal in regulating gene expression in trophoblasts, driving the establishment of trophoblast subtypes during placentation.^[^ ^26^ ^]^ Despite the remarkable progress, the principal mechanisms in STB lineage differentiation, including the critical regulatory TFs and their synergic functions, remain inadequately understood.

Cellular differentiation is characterized by dynamic expression of lineage‐specific markers, serving as identifiers of cell identity. It seems feasible to identify differentiation‐driving TFs through interrogation of direct regulators of lineage‐specific genes. Consequently, through DNA pull‐down assay of the CGB promoter sequence, we identified BHLHE40 as a pivotal regulator of human STB differentiation. Using TSCs as an in vitro model, we demonstrated that BHLHE40 expression was upregulated during STB specification. Genetic ablation of *BHLHE40* prevented STB differentiation, impairing gene expression critical for hormone synthesis and cell syncytialization. We also found that BHLHE40 activated specific sets of genes in STBs by cooperating with classical trophoblast‐lineage TFs, e.g., GATA2 and GATA3, and mediating their chromatin depositions. These findings indicate that BHLHE40 plays an indispensable role in human STB‐lineage determination, providing important insights into the molecular mechanism underlying trophoblast differentiation. Furthermore, pathological BHLHE40 reduction may underlie placental deficiencies due to STB dysfunction, providing mechanistic insights for therapeutic strategies of pregnancy complications such as miscarriage.

## 2 Results

### 2.1 CGB Promoter Pull‐Down Assay Identifies BHLHE40 as a Potential TF in Human STB Differentiation

**Figure 1:**
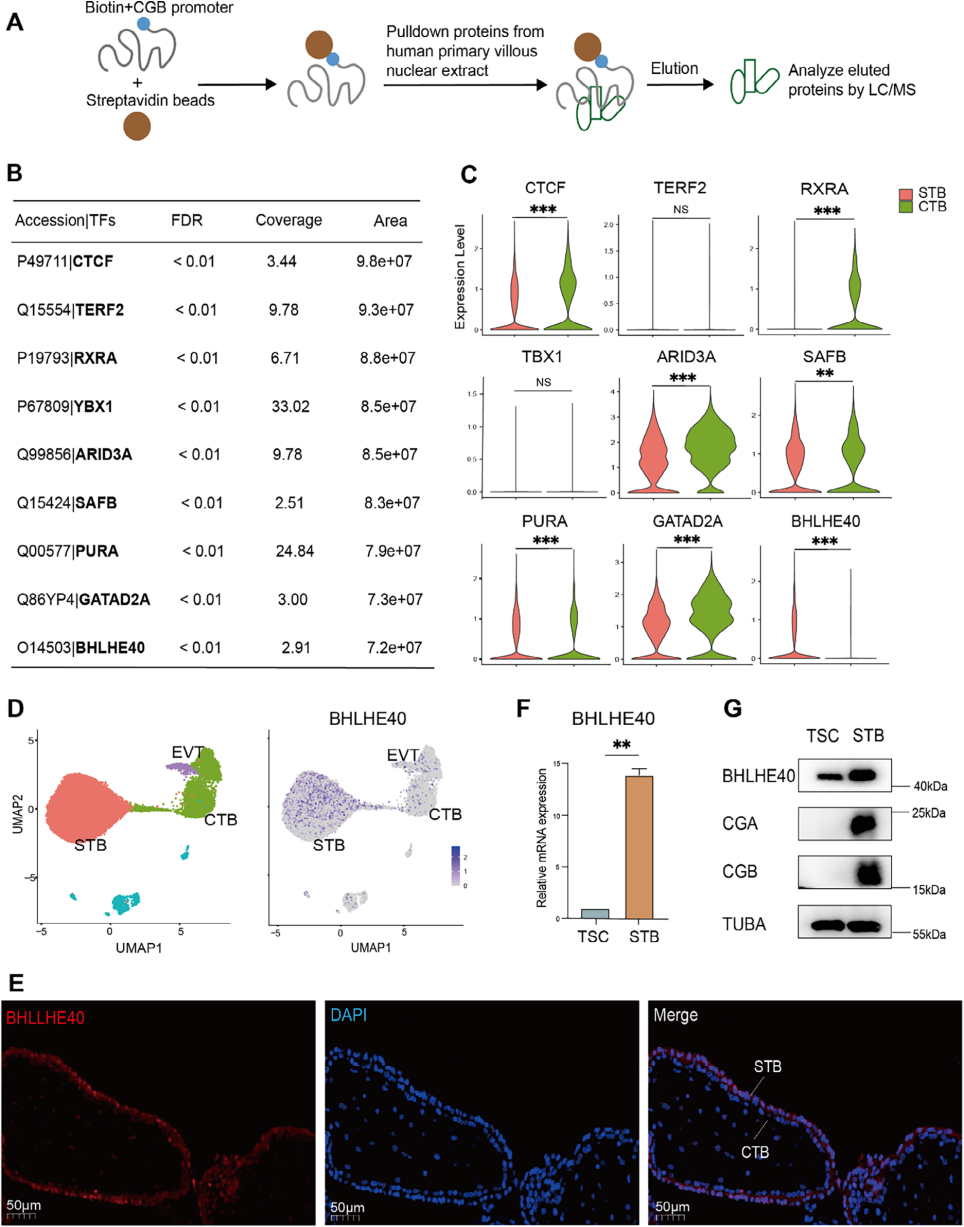
BHLHE40 is highly expressed in human syncytiotrophoblasts (STBs). A) Schematic of the pull‐down assay for the identification of proteins bound to CGB promoters. B) Top TFs identified in the pull‐down assay. C) Violin plots showing the expression levels of the listed TFs in STBs (red) and CTBs (green). ^***^ *p* < 0.001, ^**^ *p* < 0.01, NS: not significant, *p*‐values were calculated using the Wilcoxon signed‐rank test. D) UMAP showing the expression patterns of BHLHE40 for trophoblast subclusters. The expression levels are presented with color intensities. E) Immunofluorescence staining of BHLHE40 (red) in human primary villous samples obtained at 8 weeks of gestation. DAPI staining indicates nuclei (blue). F) Relative *BHLHE40* mRNA expression in human trophoblast stem cells (TSCs) and STBs. Data presented as means ± SEM, n = 3, ^**^ *p* < 0.01. *P*‐values were calculated using a two‐tailed paired Student's *t*‐test. G) Western blotting of BHLHE40 and STB markers in TSCs and STBs. Tubulin (TUBA) was used as a loading control.

To find novel regulators of STB formation, a biotin‐labeled DNA segment containing the CGB promoter sequence was immobilized onto streptavidin‐coated magnetic beads to capture proteins from the primary first‐trimester villus nuclear extract (**Figure** **1**A). The eluted proteins were analyzed by liquid chromatograph/mass spectrometry (LC/MS) analysis. From high‐confidence candidates (FDR < 0.01), the top nine TFs were selected based on relative protein abundance ranking (quantified by chromatographic peak area) (Figure 1B). (A list of all candidates is provided in Table S1, Supporting Information). Next, single‐nucleus RNA sequencing (snRNA‐seq) datasets from first‐trimester trophoblasts^[^ ^27^ ^]^ were analyzed to assess the expression level of these TFs in trophoblast subtypes. With unsupervised clustering based on canonical trophoblast markers, we found that among the nine TFs, only BHLHE40 exhibited statistically higher expression levels in STBs, compared to those in CTBs (Figure 1C). Early studies reported BHLHE40 expression in human placental tissues and its involvement in regulating EVT function.^[^ ^28^, ^29^, ^30^ ^]^ However, the potential function of BHLHE40 in syncytialization, that is, STB differentiation, remains unexplored. Based on Uniform Manifold Approximation and Projection (UMAP) visualization analysis, BHLHE40 was enriched in STBs compared to EVTs or CTBs (Figure 1D). Moreover, single‐cell regulatory network inference (SCENIC) analysis predicted that BHLHE40 was a potential driver with an increased regulatory activity for STB identity (Figure S1A, Supporting Information). The analysis also showed that genes potentially targeted by BHLHE40 were enriched in STBs (Figure S1B, Supporting Information).

Next, we validated BHLHE40 expression patterns in human placental tissues. Immunofluorescence (Figure 1E) and immunohistochemistry (Figure S1C, Supporting Information) analyses of first‐trimester villous sections revealed predominant BHLHE40 localization in STBs, with moderate expression in CTBs. It has been reported that some miscarriage cases exhibited impaired STB differentiation, characterized by reduced HCG synthesis^[^ ^31^, ^32^, ^33^, ^34^ ^]^ and syncytin‐1 expression^[^ ^35^ ^]^ in placental villi. Analysis of human villi revealed reduced BHLHE40 expression in some spontaneous miscarriages compared to normal pregnancy controls (Figure S1D, Supporting Information).

In vitro models also demonstrated BHLHE40 upregulation after STB differentiation. Both mRNA (Figure 1F) and protein (Figure 1G) levels were significantly higher in STBs differentiated from human blastocyst‐derived TSCs than in undifferentiated TSCs. Additionally, given that cAMP signaling is a key pathway in trophoblast syncytialization^[^ ^36^, ^37^ ^]^ and that cAMP response element like sequences were reported in the human *BHLHE40* promoter,^[^ ^38^, ^39^ ^]^ it seems that BHLHE40 could be a target of cAMP signaling. As expected, treatment of TSCs with db‐cAMP (1 µM) induced BHLHE40 expression (Figure S1E, Supporting Information). Furthermore, this induction was attenuated by cAMP pathway inhibitors H‐89 (PKA inhibitor; 8 µM) and ESI‐09 (EPAC antagonist; 8 µMm) (Figure S1F, Supporting Information). Together, these data suggested a potential role of BHLHE40 in STB differentiation.

### 2.2 Genome‐Wide Profiling Depicts BHLHE40 Binding Loci in Differentiated STBs

To analyze the role of BHLHE40 in STB differentiation, Cleavage Under Targets and Tagmentation (CUT&Tag) ‐seq experiments were done to examine BHLHE40 targets in cultured TSCs and STBs. Peak distribution analysis revealed comparable genomic partitioning patterns of BHLHE40 in TSCs and STBs (Figure S2A, Supporting Information). The BHLHE40 binding was predominantly in promoter regions, followed by introns and distal intergenic regions (Figure S2A, Supporting Information), indicating that BHLHE40 regulated targeted gene expression by affecting both promoter and enhancer activity. Notably, BHLHE40 exhibited comparable global binding intensity between TSCs and STBs (Figure S2B, Supporting Information). Thus, site‐specific variations in occupancy require to be further investigated.

**Figure 2:**
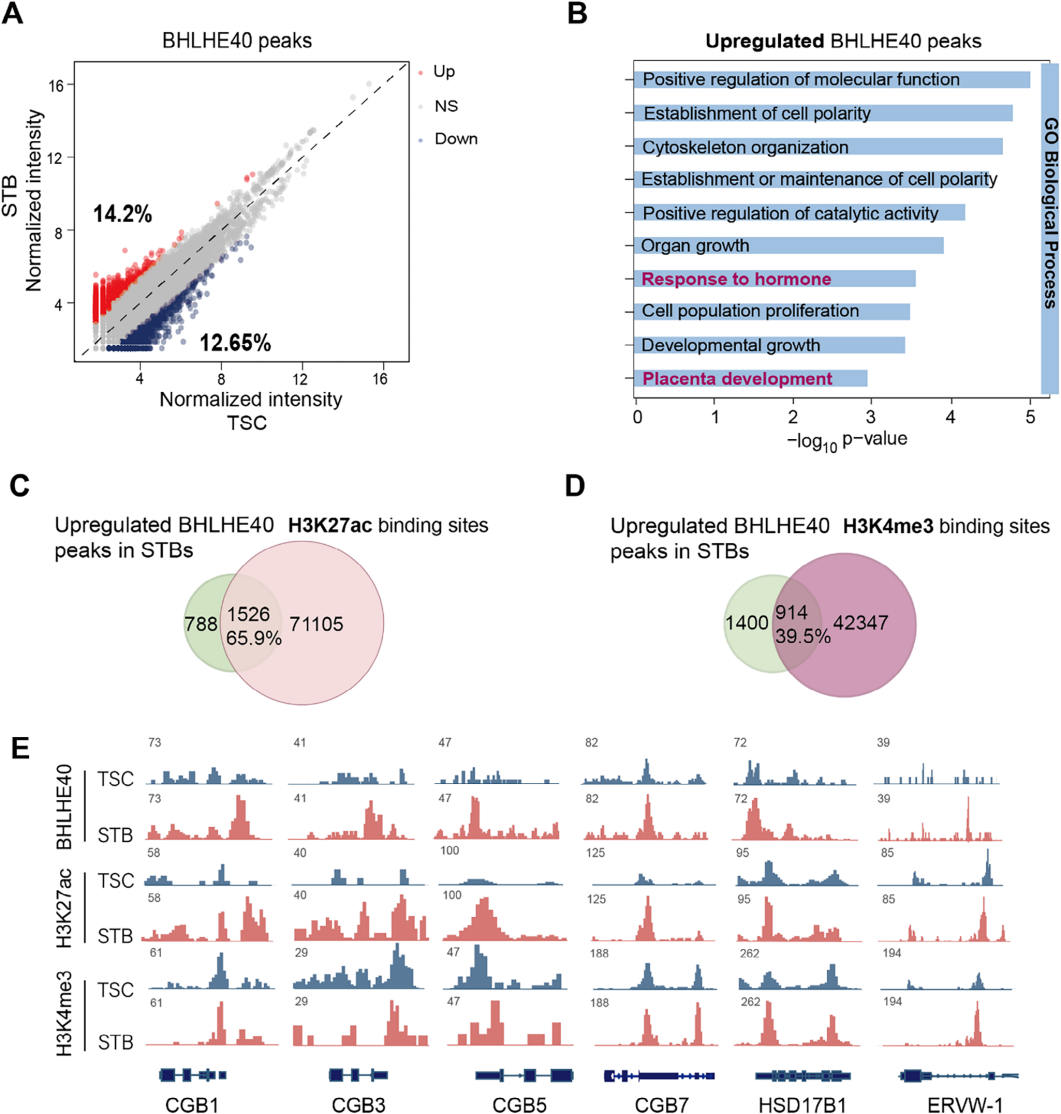
Genome‐wide profiling of BHLHE40 binding loci in STBs. A) Differential BHLHE40 CUT&Tag‐seq analysis comparing STBs versus TSCs. The sites with higher, lower, and equal signals in STBs compared with those in TSCs were represented as red, blue, and gray, respectively. Among them, 14.2% of BHLHE40 peaks were increased, and 12.65% peaks were reduced after STB differentiation. NS: not significant, *p*‐adj < 0.05. B) Selected GO terms for increased BHLHE40 peaks in STBs compared TSCs (red sites in A). C) and D) Venn diagrams showing the overlap between upregulated BHLHE40 peaks and H3K27ac (C) or H3K4me3 (D) binding sites in STBs. E) Genome browser view showing genomic peaks of BHLHE40, H3K27ac, and H3K4me3 for STB gene loci in TSCs and STBs.

Using MAnorm2 method, differential binding analysis identified peaks with upregulated and downregulated signal intensity in STBs versus TSCs (**Figure** **2**A; Figure S2C, Supporting Information). The upregulated peaks in STBs showed preferential localization to transcriptional regulatory regions, with 62% occupying intron and distal intergenic regions and 35% occupying promoter regions (Figure S2D, Supporting Information). In functional annotation through Gene Ontology (GO) analysis, genes associated with the upregulated peaks were predicted to be involved in hormone responsiveness, placental development, and cell polarity (Figure 2B). In contrast, downregulated peaks showed no functional association with STB‐related processes (Figure S2E, Supporting Information). We next carried out a conjoint analysis of differential BHLHE40 sites with two core activation‐associated histone modifications, H3K27ac and H3K4me3, both of which undergo extensive reprogramming during early embryonic development and were linked to the regulation of key gene expression.^[^ ^40^, ^41^, ^42^ ^]^ A modest global increase was found in H3K27ac and H3K4me3 signals following STB differentiation (Figure S2F, Supporting Information). Analysis revealed that 65.9% and 39.5% of upregulated BHLHE40 sites overlapped with H3K27ac and H3K4me3 binding sites in STBs, respectively (Figure 2C,D). Consistent with these findings, overlapped peaks between upregulated BHLHE40 and H3K27ac exhibited significant H3K27ac enrichment (Figure S2G, Supporting Information), whereas BHLHE40‐unique sites showed negligible signal. Similar differential enrichment patterns were observed for H3K4me3 at overlapping versus unique sites (Figure S2H, Supporting Information). In contrast, downregulated peaks in STBs had less overlaps with the H3K27ac (40.0%) and H3K4me3 (28.9%) sites (Figure S2I, Supporting Information). Furthermore, similarly increased signals were observed in STBs at BHLHE40 and H3K27ac binding loci, including key regulatory sites near *CGB*, *HSD17B1*, *ERVW‐1*, and *ERVFRDF‐1* (Figure 2E). These results suggested that BHLHE40 chromatin occupancy correlated with histone modifications associated with active gene expression during STB differentiation.

### 2.3 *BHLHE40* Knockout (KO) Prevents STB Differentiation

**Figure 3:**
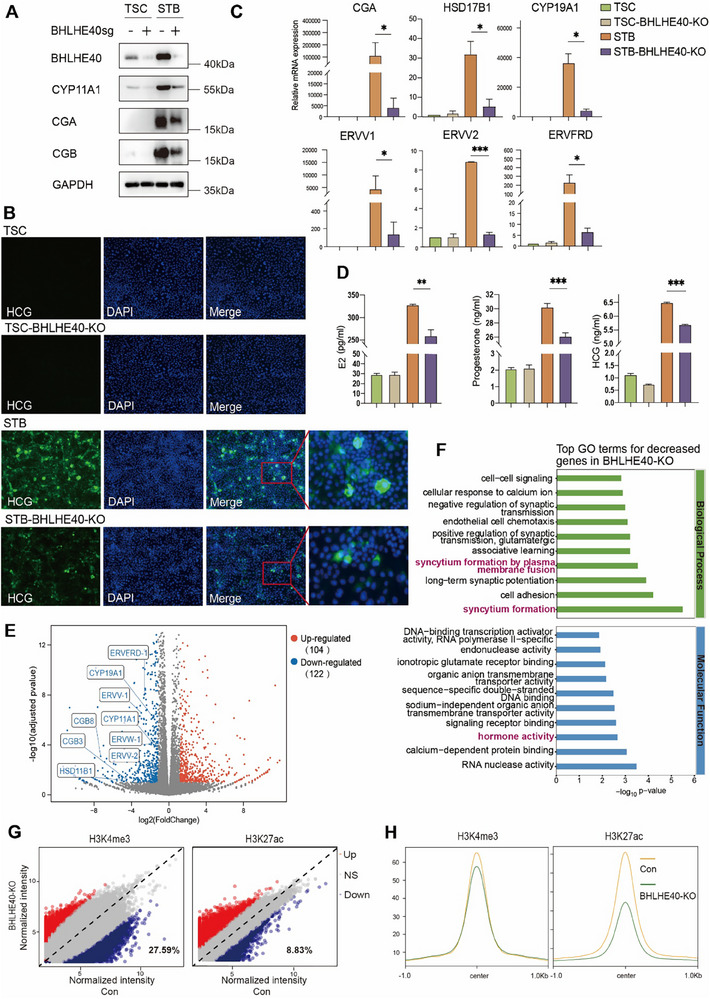
*BHLHE40* disruption impairs STB differentiation and function in culture. A) Western blotting of BHLHE40 and STB markers in wild‐type (WT) and *BHLHE40*‐KO TSCs and STBs. GAPDH was a loading control. Data shown were from KO‐1 clone (from two validated *BHLHE40*‐KO clones). B) Immunofluorescence staining of HCG (green) in WT and *BHLHE40*‐KO TSCs and STBs. DAPI indicates nuclei (blue). C) Relative mRNA expression of STB markers in WT and *BHLHE40*‐KO TSCs and STBs. Data presented as means ± SEM, n = 3, ^*^ *p* < 0.05, ^***^ *p* < 0.001. *P*‐values were calculated using a two‐tailed paired Student's t‐test. Data shown were from KO‐1 clone (selected from two validated *BHLHE40*‐KO clones). D) Levels of estrogen, progesterone, and HCG secreted by WT and BHLHE40‐KO TSCs and STBs, as measured by enzyme‐linked immunosorbent assay (ELISA). Data presented as means ± SEM, n = 3, ^**^ *p* < 0.01, ^***^ *p* < 0.001. *P*‐values were calculated using a two‐tailed paired Student's *t*‐test. E) Volcano plot depicting differentially expressed genes between WT and BHLHE40‐KO STBs. |log2FC| > 1, *p*‐adj < 0.05. Significantly up‐ and down‐regulated genes in *BHLHE40*‐KO STBs compared to WT STBs were indicated in red and blue, respectively. F) Top enriched GO terms for decreased genes in BHLHE40‐KO STBs compared to those in WT STBs. G) Differential H3K4m3 (left) and H3K27ac (right) CUT&Tag‐seq analysis comparing *BHLHE40*‐KO versus WT STBs. The sites with higher, lower, and unchanged signals in BHLHE40‐KO were in red, blue, and gray, respectively. Among them, 27.59% of H3K4me3 peaks and 8.83% H3K27ac peaks were reduced in *BHLHE40*‐KO versus WT STBs. NS: not significant, *p*‐adj < 0.05. H) Profile plots showing H3K4me3 and H3K27ac signals at highly confident BHLHE40 binding sites in WT and *BHLHE40*‐KO STBs.

To examine the importance of BHLHE40 in STB differentiation, we used CRISPR‐Cas9 to KO the *BHLHE40* gene in TSCs. *BHLHE40* disruption did not cause noticeable changes in self‐renewal or colony integrity of TSCs (Figure S3A), indicating that BHLHE40 was dispensable for the maintenance of cell stemness under the culture conditions. When wild‐type (WT) and *BHLHE40*‐KO TSCs were induced to undergo differentiation into STBs, reduced levels of CYP11A1 (related to steroidogenesis) and HCG α and β expression were detected by western blotting in STBs derived from *BHLHE40*‐KO TSCs (**Figure** **3**A). Consistently, parallel experiments using first‐trimester cytotrophoblast‐derived TSCs (TSCs^CT^) showed decreased CYP11A1, CGA, and CGB expression in *BHLHE40*‐KO STBs (Figure S3B, Supporting Information). Immunocytochemistry also showed higher levels of HCG in the STBs from WT TSCs, whereas HCG levels were lower in the STBs from *BHLHE40*‐KO TSCs (Figure 3B). In RT‐PCR, genes encoding HCG (CGA) or related to STB morphology and function (*CYP19A1*, *HSD17B*, *ERVV1*, *ERVV2*, and *ERVFRD*) were expressed at much lower levels in STBs from *BHLHE40*‐KO TSCs, compared with those from WT TSCs (Figure 3C). This transcriptional downregulation was replicated in *BHLHE40*‐KO TSC^CT^‐derived STBs for *CGA*, *CYP11A1*, *ERVV2*, *ERVFRD*, and *HSD17B1* (Figure S3C, Supporting Information), indicating BHLHE40's essential role in regulating these markers across different TSC lines. Subsequently, we assessed the impact of *BHLHE40* deficiency on syncytial fusion. WT STBs exhibited characteristic multinucleated syncytia, whereas *BHLHE40*‐KO STBs showed impaired fusion with predominant mononuclear CTB‐like cells and sparse multinucleated cells (Figure S3D, Supporting Information). In addition, reduced estrogen, progesterone, and HCG levels were found in the conditioned medium from *BHLHE40*‐KO TSC‐derived STBs, compared to those from WT TSC‐derived STBs (Figure 3D).

To examine the global transcriptomic changes, we performed RNA‐seq on WT and *BHLHE40‐*KO STBs, detecting 23910 expressed genes. Comparative analysis identified 122 significantly down‐regulated genes, many of which were STB markers, and 104 significantly up‐regulated genes in *BHLHE40*‐deficient STBs compared to WT controls (thresholds: |log2FC| > 1, *p*‐adj < 0.05) (Figure 3E). GO enrichment analysis of the down‐regulated genes indicated a close association with syncytium formation and hormone activity (Figure 3F). Reactome analysis also displayed term enrichments related to glycoprotein and steroid hormones (Figure S3E, Supporting Information). Particularly, the cAMP signaling pathway, crucial in trophoblast syncytialization,^[^ ^36^ ^]^ was enriched in the KEGG analysis of those down‐regulated genes (Figure S3F, Supporting Information). Next, CUT&Tag‐seq experiments were performed to examine BHLHE40 targets in *BHLHE40*‐KO and WT STBs. Integrative analysis revealed direct BHLHE40 binding at 54.5% of RNA‐seq‐downregulated genes, including *CG8* and *ERVV‐2* (Figure S3G, Supporting Information), suggesting a possible direct role of BHLHE40 in regulating those genes that were important in STB differentiation and function, e.g., hormone secretion.

Furthermore, CUT&Tag profiling of H3K27ac and H3K4me3 modifications revealed global alterations in these two histone activation marks in *BHLHE40*‐KO versus WT STBs. Differential peak analysis identified global reductions in both marks, with H3K4me3 exhibiting greater sensitivity (27.59%) compared to H3K27ac (8.83%) (Figure 3G). Next, differential peak analysis of BHLHE40 CUT&Tag data identified genomic loci with reduced occupancy in *BHLHE40*‐KO versus WT STBs (Figure S3H, Supporting Information). These reduced peaks were designated as highly confident BHLHE40‐binding sites, mitigating non‐specific binding artifacts. These BHLHE40‐binding loci showed significant H3K27ac attenuation but minimal H3K4me3 changes upon *BHLHE40*‐KO (Figure 3H), indicating a correlation between BHLHE40 chromatin occupancy and H3K27ac deposition. Further investigations are important to determine whether BHLHE40 directly modulates H3K27ac modification. Collectively, these data indicate BHLHE40 as a potential factor of transcription activation during STB differentiation.

### 2.4 BHLHE40 Directly Interacts with GATA2 and GATA3 in STBs

**Figure 4:**
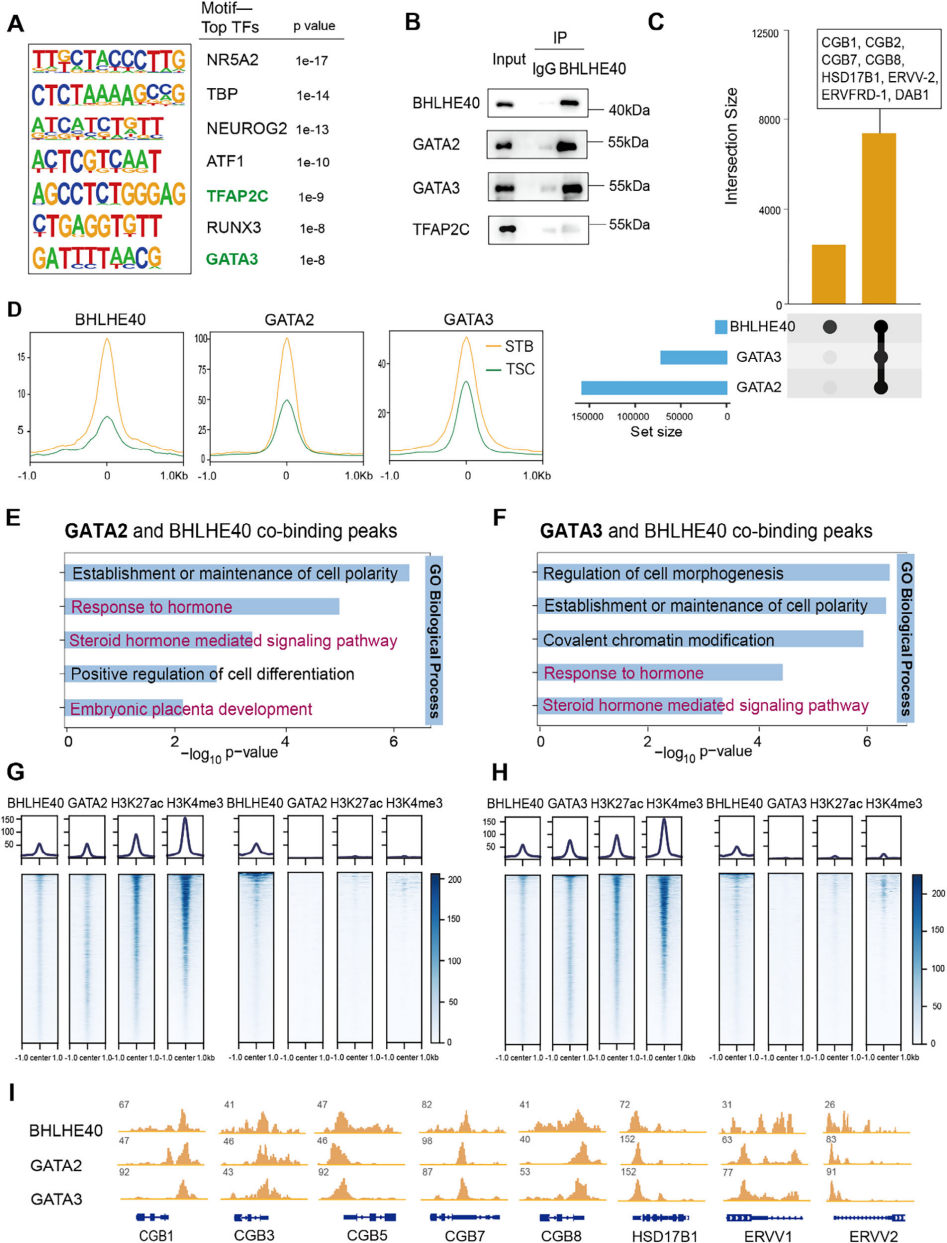
BHLHE40 interacts with GATA2/3 to form a complex that colocalizes with STB genes. A) Motifs identified by HOMER showing the top enriched DNA‐binding TFs at BHLHE40 binding sites in STBs. B) Immunoprecipitation using STB lysates with an anti‐BHLHE40 antibody and western blotting of GATA2/3 and TFAP2C. C) Upset plot depicting the overlap among peaks bound by BHLHE40, GATA2, and GATA3 in STBs. Intersection size: peak counts in specified TF combinations (black dots: included factors; gray dots: excluded). Set size: total peaks per TF. D) Profiles of BHLHE40, GATA2, and GATA3 at BHLHE40 peaks with increased signal in STBs (yellow lines) versus TSCs (green lines). E) Selected GO terms enriched for overlapping peak regions between BHLHE40 and GATA2 in STBs. F) Selected GO terms enriched for overlapping peak regions between BHLHE40 and GATA3 in STBs. G) Profile plot and heatmap showing BHLHE40, GATA2, H3K27ac, and H3K4me3 signals in BHLHE40 and GATA2 co‐binding peaks (left) and non‐overlapping BHLHE40 peaks with GATA2 (right). H) Profile plot and heatmap showing BHLHE40, GATA3, H3K27ac, and H3K4me3 signals in BHLHE40 and GATA3 co‐binding sites (left) and non‐overlapping BHLHE40 peaks with GATA3 (right). I) Genome browser view showing BHLHE40, GATA2, and GATA3 genomic enrichment peaks for representative STB gene loci in STBs.

To elucidate the role of BHLHE40 in STB differentiation, a systematic analysis of the genome‐wide BHLHE40 binding landscape was conducted. De novo motif analysis of BHLHE40‐binding regions using the HOMER method identified consensus motifs for TFAP2C and GATA3, two TFs involved in trophoblast fate‐determination (**Figure** **4**A).^[^ ^23^ ^]^ Previous studies have shown that TFAP2C can interact with YAP1 and TEAD4 to form a complex, regulating the transcription of trophoblast‐specific genes.^[^ ^43^ ^]^ GATA3 has also been found to interact with other TFs, contributing to cell lineage determination.^[^ ^44^ ^]^ To verify whether BHLHE40 directly interacts with TFAP2C and GATA3, we did co‐immunoprecipitation (Co‐IP) assays in STBs differentiated from TSCs in culture. In western blotting, both GATA3 and GATA2 (another GATA TF family member implicated in syncytialization) were detected among proteins co‐precipitated by an antibody against BHLHE40 (Figure 4B). Although TFAP2C immunoprecipitation was also observed, in comparison to the IgG control, its enrichment was much lower than that of GATA2/3 (Figure 4B). These protein‐protein interactions were verified in transfected HEK293T cells expressing HA‐tagged BHLHE40 and FLAG‐tagged GATA2 and GATA3 proteins, showing reciprocal interactions between BHLHE40 and GATA2/3 (Figure S4A,B, Supporting Information).

Next, CUT&Tag‐seq for GATA2 and GATA3 was performed in STBs and integrated with BHLHE40 chromatin occupancy data to determine whether BHLHE40 interacts with GATA2/3 to regulate gene expression. Genome‐wide co‐localization analysis identified co‐occupied loci between BHLHE40 and GATA2/3, some of which co‐localized at STB‐specific genes such as *CGB*, *HSD17B1*, and genes associated with cell fusion (Figure 4C). We analyzed GATA2 and GATA3 binding signals at genomic loci exhibiting increased BHLHE40 occupancy in STBs versus TSCs (red sites in Figure 2A). At these BHLHE40‐increased sites, we observed parallel enhancement of GATA2/3 binding signals following STB differentiation, consistent with the established protein‐protein interaction between BHLHE40 and GATA2/3(Figure 4D). Further analyses indicated that 74.6% (8794) and 69.2% (8152) of BHLHE40 peak regions overlapped with those of GATA2 and GATA3, respectively, in STBs (Figure S4C, Supporting Information). Based on GO analysis, those overlapping regions were related to hormone response, hormone signaling pathways, and cell polarity (Figure 4E,F). In contrast, unique BHLHE40 peaks that did not overlap with GATA2 or GATA3 sites (Figure S4D, Supporting Information) were not associated with functional enrichments for STB‐related processes (Figure S4E, Supporting Information). These results suggested that BHLHE40 probably interacted with GATA2/3 to regulate gene expression in STBs.

Additionally, enhanced H3K27ac and H3K4me3 signals were observed at BHLHE40‐GATA2 overlapping loci, compared to the BHLHE40 unique sites, which did not overlap with GATA2 loci (Figure 4G) in STBs. Similar results were found when BHLHE40 and GATA3 peak loci in STBs were analyzed (Figure 4H). Notably, GATA2 unique sites (Figure S4F, Supporting Information, left) and GATA3 unique sites (Figure S4F, Supporting Information, right), both of which did not overlap with BHLHE40, exhibited moderate H3K27ac and H3K4me3 signals, suggesting that BHLHE40 may be involved in H3K27ac and H3K4me3 deposition through GATA2/3 recruitment. Moreover, Integrative Genomics Viewer (IGV) visualization analysis confirmed spatial overlapping peaks of BHLHE40, GATA2, and GATA3 at *CGB1/3/5/7/8*, *HSD17B1*, *ERVV1*, and *ERVV2* loci in STBs, exhibiting similar signatures of shape and localization (Figure 4I). Together, these findings indicated that BHLHE40 directly interacted with GATA2/3, which may regulate gene expression, differentiation, and function of STBs.

### 2.5 *BHLHE40*‐KO Alters GATA2 and GATA3 Binding Patterns in STBs

**Figure 5:**
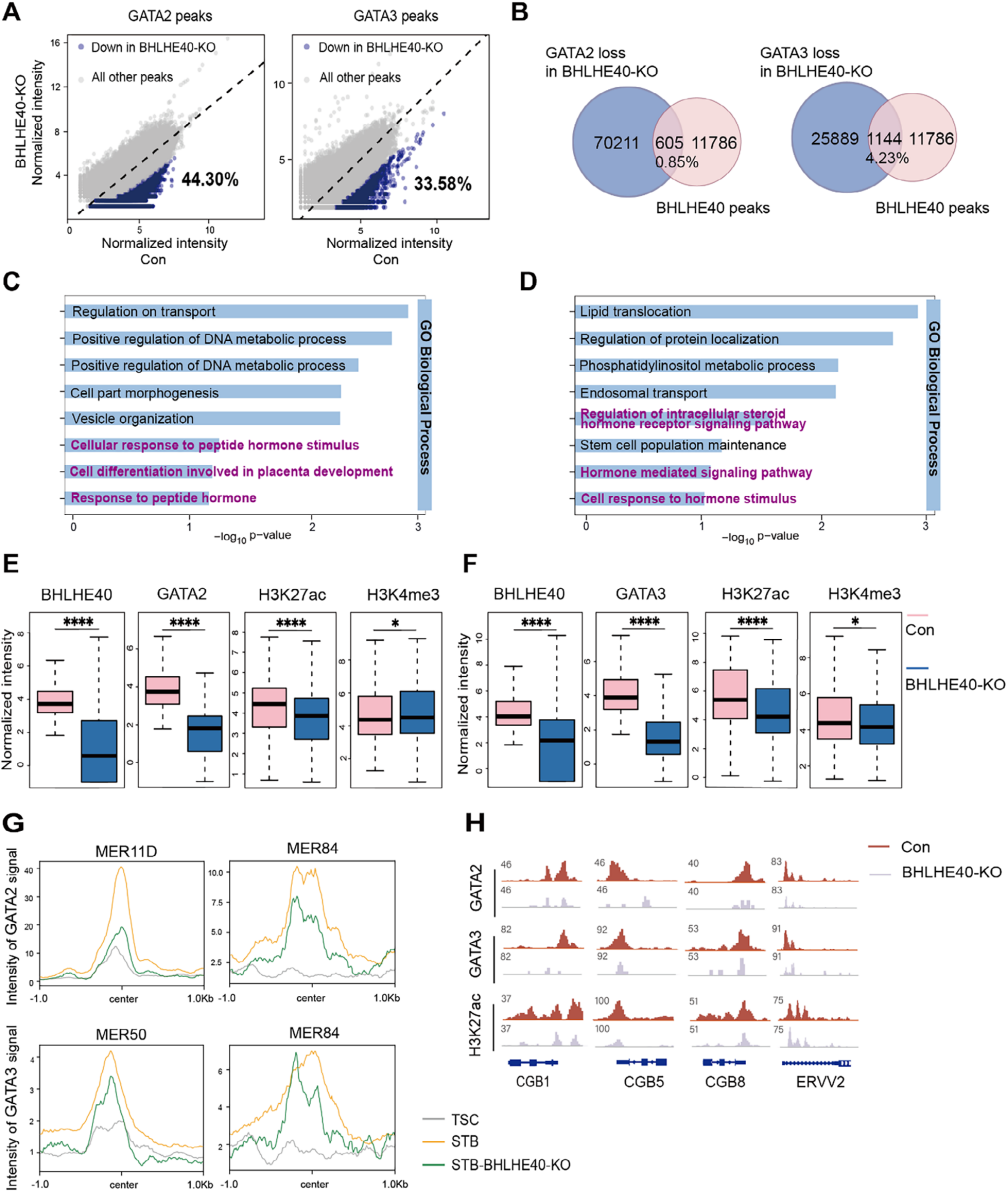
*BHLHE40*‐KO alters chromatin landscapes of GATA2/3 in STBs. A) Differential GATA2 (left) and GATA3 (right) CUT&Tag‐seq analysis comparing *BHLHE40*‐KO versus WT STBs. The sites with lower signals in *BHLHE40*‐KO were in blue, and others were in gray. Among them, 44.30% of GATA2 peaks and 33.58% GATA3 peaks were reduced in *BHLHE40*‐KO versus WT STBs. *p*‐adj < 0.05. B) Venn diagram showing the overlap between GATA2/3 loss in *BHLHE40*‐KO and BHLHE40 peaks in STBs. C) and D) Selected GO terms enriched for overlapped peaks between GATA2 (C) or GATA3 (D) loss in *BHLHE40*‐KO and BHLHE40 peaks in STBs. E) and F) Boxplots showing the normalized intensity of BHLHE40, GATA2/3, H3K27ac, and H3K4me3 signals at the BHLHE40‐dependent downregulated GATA2 (E) and GATA3 (F) binding sites in WT versus *BHLHE40*‐KO STBs. ^****^ *p* < 0.0001, ^*^ *p* < 0.05. *P*‐values were calculated using the Wilcoxon signed‐rank test. G) Profiles of GATA2 (top panel) or GATA3 (bottom panel) signals at ERV subtypes associated with STB genes in WT and *BHLHE40*‐KO STBs. H) Genome browser view showing GATA2, GATA3, and H3K27ac genomic enrichment peaks for representative STB gene loci in WT and *BHLHE40*‐KO STBs.

To examine the potential effects of *BHLHE40* KO on GATA family binding landscape and chromatin remodeling, the CUT&Tag‐seq was performed for GATA2 and GATA3 in *BHLHE40*‐KO STBs. Differential binding analysis revealed 44.30% (70816) of GATA2 and 33.6% (27033) of GATA3 peaks exhibited significant signal reduction in *BHLHE40*‐KO STBs compared to WT STBs (**Figure** **5**A). Notably, the binding signals of GATA2/3 at these attenuated peak sites exhibited an upward trend following normal STB differentiation (Figure S5A,B, Supporting Information), which indicated that BHLHE40‐affected GATA2/3 loci likely represent important regulatory elements for STB differentiation. Among the reduced peaks, 605 GATA2 and 1144 GATA3 sites overlapped with BHLHE40‐binding loci (Figure 5B), suggesting that BHLHE40 deficiency might directly affect GATA2/3 binding profiles at these specific genomic regions in STBs. Further GO analysis indicated that these BHLHE40 and GATA2 (Figure 5C) or GATA3 (Figure 5D) loss overlapping sites were enriched in cellular processes associated with STB differentiation, e.g., responses to hormones, protein localization, and cell differentiation. Genomic distribution analysis showed BHLHE40‐dependent downregulated GATA2 peaks localized preferentially to intron (37%), distal intergenic (31%), and promoter (29%) regions, with analogous partitioning observed for GATA3 (Figure S5C, Supporting Information). These results suggested that BHLHE40 deficiency may alter GATA2/3 binding to promoters as well as enhancers at distal genomic regions.

To test this hypothesis, further CUT&Tag analysis was performed for the BHLHE40‐dependent decreased GATA2/3 peaks in *BHLHE40*‐KO STBs using WT STBs as a control. Normalized signal quantification indicated that the sites of GATA2 (Figure 5E) or GATA3 (Figure 5F) signal loss were accompanied by a marked reduction in H3K27ac and, to a lesser degree, H3K4me3 peaks (Figure 5E,F). Previously, endogenous retrovirus (ERV)‐derived enhancers were found as transcriptional regulators in human trophoblast syncytialization.^[^ ^14^ ^]^ These genomic elements with long terminal repeat sequences contributed to the evolution of mammalian placentas.^[^ ^45^, ^46^, ^47^ ^]^ We analyzed a set of ERV families that are located near STB genes and highly enriched at STB enhancers.^[^ ^14^ ^]^ The GATA2 signal peaks at *MER11D* and *MER84* increased when TSCs were differentiated into STBs, but such an increase was attenuated in the *BHLHE40*‐KO STBs (Figure 5G, top panel). Similar results were found in GATA3 at *MER50* and *MER84* (Figure 5G, bottom panel), suggesting that BHLHE40 played a unique role in GATA2/3 binding at these loci. GATA2/3 binding signals at remaining ERV family subtypes exhibited no significant changes upon BHLHE40 loss (Figure S5D, Supporting Information). Moreover, GATA2/3 and H3K27ac peaks at *CGB1/5/8* and *ERVV2* loci were significantly reduced in *BHLHE40*‐KO STBs, compared to control WT STBs (Figure 5H). These results suggested that BHLHE40 might alter GATA2 and GATA3 interactions with other genomic elements to regulate gene expression and differentiation of STBs.

## 3 Conclusion

**Figure 6:**
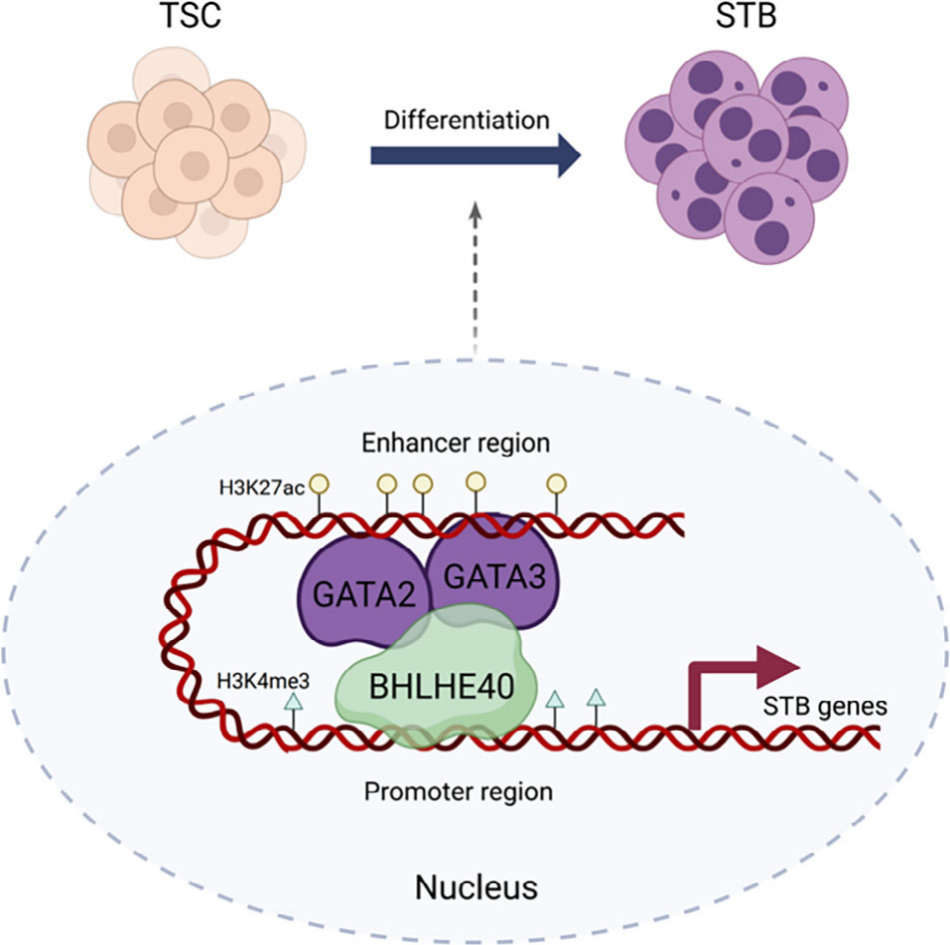
Proposed diagram of BHLHE40 cooperating with GATA2/3 to regulate STB differentiation. BHLHE40 interacts directly with GATA2 and GATA3 and co‐occupies genomic loci critical for STB differentiation and function. BHLHE40 also affects GATA2/3 binding at enhancer and promoter regions. Moreover, BHLHE40 is potentially involved in genome‐wide remodeling of H3K4me3 and H3K27ac chromatin landscapes through GATA2/3 recruitment, activating transcriptional programs of STB genes. Possibly, this proposed transcriptional machinery may drive TSCs to differentiate into multinucleated STBs.

Placental development relies on proper differentiation of CTBs into multinucleated STBs and invasive EVTs. Here, we identified BHLHE40, a multifunctional TF, as a crucial regulator in human STB differentiation. BHLHE40 expression was increased during STB differentiation but reduced in villous from patients with miscarriages. Knockout of the *BHLHE40* gene prevented human TSCs from differentiating into STBs in culture. Mechanistically, BHLHE40 interacted directly with GATA2/3 to form a complex that localized at STB‐related genes. Furthermore, BHLHE40 loss impaired GATA2/3 chromatin occupancy at promoter and distal enhancer regions, which might disrupt transcriptional activation of differentiation programs in STBs. Collectively, our study uncovered a BHLHE40‐GATA2/3 regulatory network governing human STB lineage determination and HCG production (**Figure** **6**).

Multiple TFs have been identified that independently or cooperatively regulate gene expression in STBs,^[^ ^47^ ^]^ indicating that CTB differentiation depends on a regulatory network of TFs. However, the critical members of the TF network and their synergic functions in STB lineage specification remain inadequately understood. Here, through delineating the expression pattern of BHLHE40 during trophoblast lineage differentiation, we found that BHLHE40 was upregulated after syncytialization. Activation of cAMP signaling likely mediated elevated BHLHE40 expression in STBs. Consistently, BHLHE40 has been reported to regulate gene expression in cells activated by cAMP signaling,^[^ ^38^ ^]^ which prompted our interest in its transcriptional regulatory role in STBs. Additional studies are required to determine the exact molecular mechanism and signaling pathways that regulate BHLHE40 function at trophoblast differentiation stages. Our study showed that *BHLHE40*‐KO in TSCs inhibited STB differentiation and expression of STB‐related genes. In *BHLHE40*‐KO STBs, secretion of estrogen, progesterone, and HCG was reduced, mirroring the hormonal deficiency in miscarriage patients. Considering our findings of reduced BHLHE40 levels in first‐trimester villous samples from women with miscarriages, the decline in HCG levels in these patients might be, at least in part, due to low levels of BHLHE40. Therefore, BHLHE40 deficiency in STBs may contribute to placental insufficiency, potentially leading to adverse pregnancy outcomes. Further investigation of its mechanistic involvement in pregnancy complications is important. A recent study demonstrated that Bhlhe40 overexpression prevented benz[a]anthracene‐induced fetal loss in mice, attenuating the toxicant's detrimental effects on placental function.^[^ ^29^ ^]^ Conversely, aberrant BHLHE40 upregulation in third‐trimester preeclamptic placenta was reported, with murine models showing symptom alleviation following *Bhlhe40* knockdown.^[^ ^48^ ^]^ These findings suggested a potential gestation‐stage‐specific role of BHLHE40, warranting further investigation of pathological regulatory mechanisms. Elucidating the molecular pathophysiology of these pregnancy complications may provide new insights for developing therapeutic strategies.

In this study, we proposed a screening strategy to find TFs that regulate lineage specification through analysis of the binding proteins onto the promoter regions of cell identity‐defining genes. This strategy can be extended to screen for regulatory TFs in other cell differentiation processes. Through the pull‐down assay using the *CGB* promoter, we identified BHLHE40 as a potential TF in regulating STB differentiation. BHLHE40 binds to the conserved CACGTG motif in the DNA sequence. While known as a transcriptional repressor via histone deacetylases ‐dependent and independent mechanisms,^[^ ^49^ ^]^ BHLHE40 also activates transcription.^[^ ^50^, ^51^ ^]^ Studies have shown that BHLHE40 is critical in immune regulation, including inflammation, infection, and autoimmunity.^[^ ^52^ ^]^ Moreover, BHLHE40 also participates in other processes, including circadian rhythm, hypoxia, and carcinogenesis.^[^ ^53^, ^54^, ^55^ ^]^ Early studies have detected BHLHE40 expression in human first‐trimester villous explant‐derived EVTs and the EVT‐derived cell line HTR‐8/Svneo.^[^ ^30^, ^56^ ^]^ BHLHE40 was also reported to promote human trophoblast proliferation and migration, suggesting its potential role in EVT differentiation.^[^ ^29^, ^30^ ^]^ However, its expression pattern in human primary STBs and regulatory role in STB fate commitment remain undefined. Our work extends the knowledge regarding the functional repertoire of BHLHE40. In good agreement with our findings, a recent CRISPR screening in human TSCs identified a set of genes implicated in STB differentiation, with *BHLHE40* included, albeit not identified as a top candidate under the screening conditions and therefore lacked subsequent functional validation.^[^ ^57^ ^]^ Investigations into mouse trophoblast development revealed detectable *Bhlhe40* mRNA levels during in vitro TSC differentiation, with possible roles of Bhlhe40 in promoting trophoblast giant cell differentiation.^[^ ^58^, ^59^ ^]^ Notably, despite documented immune dysfunction in *Bhlhe40*‐deficient mice,^[^ ^51^, ^60^ ^]^ reproductive abnormalities were not reported. Future investigations should examine the spatiotemporal expression profile of Bhlhe40 in mouse placenta and determine whether its deficiency disrupts trophoblast differentiation and thus leads to pregnancy failure.

Cell fate determination is governed by intertwined genetic and epigenetic networks, encompassing transcriptional reprogramming and chromatin remodeling.^[^ ^61^, ^62^ ^]^ GATA3 is expressed across trophoblast lineages, from blastocyst‐stage trophectoderm (TE) to terminally differentiated trophoblasts.^[^ ^63^ ^]^ The differentiation of TSCs into STBs may rely on the coordinated function of GATA3 together with its homologous gene GATA2.^[^ ^24^ ^]^ Also, GATA2 and GATA3 regulated the transcription of the EVT‐specific marker HLA‐G via the enhancer L.^[^ ^64^ ^]^ It appears that GATA2 and GATA3 may act in separate trophoblast subtypes via distinct molecular mechanisms to mediate cell fate determination. We showed that *BHLHE40*‐KO disrupted genome‐wide chromatin occupancies of GATA2/3 in STBs, reducing their binding at multiple loci related to STB functional genes. These results indicated a potential role of BHLHE40 in stabilizing the genomic binding of the GATA family. Possibly, BHLHE40, enriched in STBs, could recruit GATA2/3 to promote the expression of the targeted genes in STBs. In line with this hypothesis, diminished GATA2 and GATA3 binding signals, including those in enhancer regions, were observed in *BHLHE40*‐KO STBs. The GATA family can recruit acetyltransferases (e.g., CBP/p300) to shape local histone acetylation landscapes.^[^ ^65^, ^66^ ^]^ Therefore, the signal loss of GATA2/3 provided a possible explanation for the reduced H3K27ac peaks observed following BHLHE40 loss. To date, no evidence supports intrinsic acetyltransferase activity in BHLHE40 or direct recruitment of acetyltransferases for chromatin modification, with only one study reporting an interaction between BHLHE40 and P/CAF (p300/CBP‐associated factor).^[^ ^67^ ^]^ In our study, H3K27ac enrichment was observed only at BHLHE40‐GATA2/3 co‐occupied loci, with negligible signal at BHLHE40 unique sites in STBs. Possibly, BHLHE40 might regulate H3K27ac modification indirectly through GATA2/3 interactions rather than direct recruitment of acetyltransferases in STBs. Further studies are needed to understand the molecular mechanisms in the recruitment and complex formation of BHLHE40, GATA2, and GATA3 in the context of chromatin modification in STBs.

Extensive studies have established critical immunological functions for both BHLHE40 and GATA3. GATA3 orchestrates adaptive immunity by regulating T cell development, proliferation, and maintenance.^[^ ^68^ ^]^ It serves as a master regulator of T helper 2 (Th2) cell differentiation^[^ ^69^ ^]^ and is essential for regulatory T (Treg) cell homeostasis and function.^[^ ^70^ ^]^ Although BHLHE40 is dispensable for lineage‐specific gene expression in Th cells, it governs cytokine production. Compared to WT cells, Bhlhe40^−/−^ Th1, Th2, and Th17 lineages exhibited reduced GM‐CSF but elevated IL‐10 production.^[^ ^71^, ^72^ ^]^ Besides, BHLHE40 maintained Treg homeostasis and promoted their survival and expansion.^[^ ^51^ ^]^ These findings suggested potential functional overlap between GATA3 and BHLHE40 in immune regulation. Notably, a previous study has reported that GATA3 maintained Th17 responses through BHLHE40 and GM‐CSF modulation.^[^ ^73^ ^]^ Our study thus may provide mechanistic insights into immune cell subset differentiation and maintenance. Future investigations should help to determine whether BHLHE40‐GATA3 interactions occur in extraplacental systems and explore their potential epigenetic coordination within immune networks or other physiological contexts.

In conclusion, our study reveals a regulatory role of BHLHE40 in human STB differentiation. Specifically, BHLHE40 interacts with GATA2/3 and affects their chromatin occupancy at transcriptional regulatory regions, thereby enhancing transcription programs critical for STB lineage determination. These findings uncover regulatory mechanisms of trophoblast differentiation and provide important insights into potential therapeutic strategies for pregnancy disorders associated with placental dysfunction.

## 4 Experimental Section

### Human TSC Culture and STB Differentiation

TSC lines derived from human blastocysts (B7) and the first‐trimester cytotrophoblasts (CT11) were generously provided as a gift by Dr. Hiroaki Okae (Department of Informative Genetics, Environment and Genome Research Center Tohoku University Graduate School of Medicine). TSCs were cultured in Matrigel‐coated (354277, Corning) plates and maintained in Advanced DMEM/F‐12 medium with 0.2% fetal bovine serum (FBS), 0.3% bovine serum albumin (BSA; Sigma–Aldrich, A9418), 1% ITS‐X (Gibco, 51500), 50 ng mL^−1^ recombinant human epidermal growth factor (EGF; Peprotech, AF‐100‐15), 2 µMm CHIR99021 (TargetMol, T2301), 0.5 µMm A83‐01 (PeproTech, 90943360), 5 µM Y‐27632 (TargetMol, T1870), 1 µMm SB431542 (TargetMol, T1726), 0.8 mMm valproic acid (TargetMol, T7064), 1.5 mg mL^−1^ L‐ascorbic acid (TargetMol, T0928), 0.1 mMm β‐mercaptoethanol (Thermo Fisher Scientific, 21985023), and 0.5% penicillin‐streptomycin. For cell passages, TSCs were washed with phosphate‐buffered saline (PBS) and dissociated by TrypLE Express (Thermo Fisher Scientific) for 5 min, followed by seeding at a 1:3 to 1:4 ratio.

For STB differentiation, TSCs were plated on Matrigel‐coated plates. Upon cell adhesion, the culture medium was replaced with differentiation medium consisting of Advanced DMEM/F‐12 with 0.3% BSA, 4% KnockOut Serum Replacement (KSR; Thermo Fisher Scientific, 10828028), 1% ITS‐X, 5 µM Y‐27632, 1 µM dibutyryl cyclic AMP (db‐cAMP), 2 µM forskolin (FSK), and 0.5% penicillin‐streptomycin. Differentiated STBs were harvested after 48 h.

### Generation of BHLHE40‐KO TSCs

A single‐guide RNA (sgRNA) sequence (CAAGTGTACAAGTCAAGACG) targeting *BHLHE40* was cloned into the piggyBac‐EF1A‐Cas9‐U6‐sgRNA‐PuroR plasmid. TSCs were transfected with 2 µg of the Cas9‐gRNA‐expressing plasmid and 1 µg piggyBac transposase‐expressing plasmid using polyethylenimine (PEI) according to the manufacturer's instructions. Selection of transfected cells was done using puromycin (5 µg mL^−1^) starting three days post‐transfection. Surviving cells were dissociated into single cells and cultured at low density. Colonies from single cells were isolated, expanded, and verified by western blotting. Two independently verified BHLHE40‐KO TSC clones (designated KO‐1 and KO‐2) were utilized for subsequent experiments, with representative data from clone KO‐1 shown in figures. Phenotypic consistency was confirmed in both clones.

### Isolation of Human Primary Villous Tissues

This study received approval from the Human Research Ethics Committee of Obstetrics and Gynecology Hospital, Fudan University (No. kyy2023‐17). Written informed consent was obtained from all participants prior to tissue collection. Human placental villous tissues were from first‐trimester pregnant women (gestational age: 6–10 weeks) aged 18–35 years, including those of elective termination with no prior miscarriage history and spontaneous miscarriages based on the absence of fetal cardiac activity and/or abnormal gestational sac development. Villous tissues were dissected from the maternal uterine tissue and rinsed with ice‐cold PBS to remove blood. For protein extraction, tissues were homogenized in radioimmunoprecipitation assay (RIPA) buffer (50 mM Tris‐HCl, pH 7.4, 150 mM NaCl, 0.1% SDS, 1% sodium deoxycholate, and 1% Triton X‐100) with a protease inhibitor cocktail (1x; MCE) and nuclease (Beyotime, China) at a ratio of 100 mg tissue per 1 mL buffer. Homogenates were put on ice for 30 min and centrifuged at 12000 × g for 30 min at 4 °C. The supernatant was collected for further analyses.

### DNA Pull‐Down

A biotinylated DNA probe spanning the human CGB promoter region (1000 bp upstream of the transcription start site) was generated by PCR amplification using primers listed in Table S2 (Supporting Information). The biotinylated probe was incubated with streptavidin‐coated magnetic beads at room temperature (RT) for 1 h. Nuclear extracts were prepared from 2–3 human first‐trimester villus using hypotonic buffer (20 mM Tris‐HCl, pH 7.5, 10 mM NaC1, and 0.1% NP‐40) with rotation at RT for 15 min and then lysed using buffer containing 20 mM Tris‐HCl, pH 7.5, 150 mM NaCl, and 0.5% Triton‐X100 with rotation at 4 °C overnight. The lysates were centrifuged and incubated with the aforementioned DNA‐bead complexes at 4 °C for 4 h with rotation. After incubation, sequential washes were done using low‐ (20 mM Tris‐HCl, pH 7.5, 150 mM NaCl, and 0.1% NP‐40) and high‐ (20 mM Tris‐HC1, pH 7.5, 300 mM NaCl, and 0.1% NP‐40) salt buffer (three washes per buffer condition) to remove nonspecific binding proteins. Captured proteins were eluted with Benzonase (Yeason, China) and subjected to SDS‐PAGE, followed by LC‐MS/MS analysis. Mascot and MaxQuant bioinformatics platforms were used to obtain the candidate list.

### snRNA‐seq Data Analysis

snRNA‐seq data from human villous tissue were from public datasets (https://Figureshare.com/s/ccec028e5a3ec5b20106).^[^ ^27^ ^]^ Quality control and gene quantification were performed to retain high‐quality cells. The function FindVariableGenes (mean.function = FastExpMean, dispersion.function = FastLogVMR) from the Seurat package (v4.0.0) was used to identify the top hypervariable genes (HVGs). Principal component analysis (PCA) was conducted via the RunPCA function for dimensionality reduction. Cell clusters were generated based on HVG expression profiles using the FindClusters function and visualized via UMAP. Trophoblast subpopulations were annotated by computing correlations between cellular expression profiles and canonical trophoblast markers using the SingleR package (v1.4.1).^[^ ^74^ ^]^

SCENIC analysis was done following the established framework,^[^ ^75^ ^]^ which integrates co‐expression networks, cis‐regulatory motif enrichment, and regulon activity scoring. The workflow began with co‐expression network construction using GRNBoost2, which computed pairwise associations between all TFs and genes based on the normalized snRNA‐seq expression matrix from trophoblast clusters after batch correction with Seurat V4. BHLHE40‐specific interactions were retained only if they met two statistical criteria: an importance score > 3 (quantifying association strength) and classification within the top 5% of all TF‐gene interactions to prioritize high‐confidence links. Next, motif enrichment validation was conducted using RcisTarget with the hg38 database, which scanned conserved motifs in ±10 kb promoter regions. Co‐expression modules were pruned to retain genes exhibiting both co‐expression with BHLHE40 and direct binding potential, as evidenced by a Normalized Enrichment Score (NES) > 3 for BHLHE40‐binding motifs and an area under the curve (AUC) threshold > 0.85. This dual‐evidence approach ensured that only genes with robust regulatory relationships were incorporated into high‐confidence regulons. Finally, regulon activity scoring was performed using AUCell, which quantified BHLHE40 regulon activity in each cell by ranking the expression of its validated target genes.

### CUT&Tag Library Preparation and Data Analysis

CUT&Tag was performed following the protocol by Henikoff.^[^ ^76^ ^]^ Briefly, cells were dissociated using Accutase (A1110501, Gibco) at 37 °C for 5 min, washed, and incubated with Concanavalin A‐coated beads (Biolinkedin, L‐1017, China) at RT for 10 min. Cells were then incubated with the primary antibody at 4 °C overnight, followed by 1‐h incubation with an unconjugated secondary antibody (Jackson ImmunoResearch) and 1‐h incubation with pAG‐Tn5 (Novoprotein, M059‐YH01‐01A, China), preloaded with mosaic end adapters. Between incubations, beads bound to cells were washed with buffer. Tagmentation was performed by treating cell‐bound beads with MgCl_2_ at 37 °C for 1 h, generating DNA fragments. The fragments were purified using DNA extraction beads (Beyotime, China) according to the manufacturer's protocol. The purified DNA fragments were amplified by PCR (21 cycles) using 5x Multiplex PCR Mix (Novoprotein, E086‐YSAA‐01A) to prepare the CUT&Tag library.

For data analysis, sequencing libraries were processed on a NovaSeq 6000 system, with raw reads filtered and trimmed using Trim Galore (v0.6.10) and aligned to the human reference genome (hg38) using Bowtie2 (v2.3.5).^[^ ^77^ ^]^ Duplicate reads were removed using Sambamba (v0.6.6), and peak calling was performed with MACS2 (v2.2.6).^[^ ^77^ ^]^ Differential peak analysis was conducted using MAnorm2 (v1.2.2) or the DiffBind package (v3.4.11).^[^ ^78^ ^]^ Scatter plots representing differentially bound regions were generated using ggplot2 (v3.4.4) in R (v4.1.2). Motif analysis was conducted using HOMER (v4.9.8) with the findMotifsGenome.pl tool under default parameters. Peak file processing and co‐binding analysis were performed using bedtools (v2.27.1)^[^ ^79^ ^]^ and DeepTools (v3.4.5). Peak annotation and functional enrichment analyses were carried out using the ChIPseeker (v1.30.3) and ClusterProfiler (v4.2.2) packages in R. Genome browser views of binding peaks were visualized using IGV (v2.17.4).

### Reverse Transcription Quantitative PCR (RT‐qPCR)

Total RNAs were extracted using the EZ‐press RNA Purification Kit (EZ Bioscience) according to the manufacturer's protocol. Complementary DNA (cDNA) synthesis was performed using a reverse transcription kit (Yeason, China), and cDNAs were diluted prior to analysis. qPCR was conducted using a SYBR Green‐based qPCR kit (Yeason, China) on an Applied Biosystems QuantStudio 6 instrument. Relative mRNA expression levels were quantified using β‐actin (ACTB) as a control, with gene expression changes calculated as fold‐change relative to ACTB. Primer sequences are listed in Table S2 (Supporting Information).

### RNA‐seq Library Preparation and Data Analysis

All RNA samples were extracted using RNAiso reagent (TaKaRa) and exhibited RNA integrity numbers of 10.0, confirming optimal RNA integrity. Indexed libraries were prepared using the TruSeq Stranded Total RNA Library Preparation Kit (Illumina) following the manufacturer's protocol. Libraries were pooled and sequenced on a NovaSeq 6000 platform. Raw sequencing data were processed using HISAT2 (v2.0.4), including quality filtration, trimming, and alignment to the human reference genome. Gene expression levels were quantified using StringTie (v1.3.0). Differential gene expression analysis was performed using DESeq2 (v1.24), identifying differentially expressed genes (DEGs) based on *p*‐adj < 0.05 and |log2FC| > 1. Volcano plots were generated from DESeq2 results using the EnhancedVolcano package (v1.8.2) in R. Pathway enrichment analysis was performed using ClusterProfiler (v4.2.2).

### Immunohistochemistry

Fresh human villi tissues obtained at 8 weeks of gestation were washed with PBS and fixed in 4% paraformaldehyde (Biosharp). Following paraffin embedding, tissues were sectioned into 3 µm‐thick slices. Sections were incubated at 60 °C for 2 h for tissue rehydration, followed by deparaffinization and dehydration through an ethanol gradient (50%, 75%, 85%, 95%, and 100%). Antigen retrieval was performed by heating the slides immersed in citrate buffer or Tris‐EDTA (pH 9.0) and cooling to RT. Endogenous peroxidase activity was quenched by incubation with 3% hydrogen peroxide for 10 min, followed by permeabilization using PBST for 20 min. To block nonspecific binding, sections were incubated with 5% normal goat serum for 30 min at RT, then probed with primary antibody (anti‐BHLHE40, ABclonal; A6534, diluted according to the manufacturer's instructions) at 4 °C overnight. The slides were incubated with secondary antibodies at RT for 30 min. Chromogenic detection was carried out using a DAB kit and counterstained with hematoxylin for 1 min. Following dehydration in an ascending ethanol gradient (50%, 75%, 85%, 95%, 100%) and xylene, the sections mounted with a neutral polymer medium and visualized under optical microscopy.

### Western Blotting

Cell lysates were prepared using RIPA buffer (50 mM Tris‐HCl, pH 7.4, 150 mM NaCl, 0.1% SDS, 1% sodium deoxycholate, and 1% Triton X‐100) with a protease inhibitor mixture (TargetMol, C0001) and Benzonase (Yeason, China). Protein concentrations were determined using a BCA assay, and 10 µg of each protein sample was separated by 7.5% or 10% SDS‐PAGE and transferred to PVDF membranes (0.45 µm pore size). The membranes were blocked with 5% skim milk in TBST at RT for 1 h, followed by incubation with primary antibodies at 4 °C overnight. The primary antibodies included: anti‐BHLHE40 (Abcam; ab259837), anti‐CGA (ABclonal; A1239), anti‐CGB (ABclonal; A12419), anti‐CYP11A1 (ABclonal; A23954), anti‐GATA2 (Abcam; ab109241), anti‐GATA3 (Abcam; ab199428), anti‐TFAP2C (Abcam; ab218107), anti‐DDDDK tag (Abcam; ab205606), anti‐HA tag (Abcam; ab9110), anti‐tublin (Beyotime; AF2827), anti‐GAPDH (Abcam; ab8245), and anti‐β‐actin (Abcam; ab8226), all of which were diluted according to the manufacturer's instructions. After washing, the membranes were incubated with horseradish peroxidase (HRP)‐conjugated secondary antibodies: goat anti‐rabbit IgG (Proteintech; SA00001‐2) or goat anti‐mouse IgG (Proteintech; SA00001‐1) at RT for 1 h. Protein bands were visualized using GE Healthcare Amersham Imager 600 system.

### Coimmunoprecipitation (Co‐IP)

Cells were harvested from culture plates and lysed in hypertonic buffer (20 mM Tris‐HCl pH 7.5, 300 mM NaCl, and 0.1% Triton X‐100), with a protease inhibitor mixture and Benzonase (Yeason, China) for 10 min at RT with gentle rotation. The samples were centrifuged, and the supernatants were collected.  Protein concentrations were determined using a BCA assay. 50 µg of each sample was reserved as input; lysate equivalent to 1 mg protein was incubated with protein A/G magnetic beads (Yeason) pre‐conjugated with primary antibodies, followed by overnight rotation at 4 °C. The primary antibodies used in this experiment were anti‐BHLHE40 (Santa Cruz; sc‐101023), anti‐DDDDK tag (Abcam; ab205606), and anti‐HA tag (Abcam; ab9110), with 1–2 µg antibody per 1 mg of total protein. To remove unbound proteins, the samples were loaded onto an Invitrogen magnetic separator to isolate the antibody‐bound protein complexes, followed by washing five times with TBST. Proteins were eluted using a 1*loading buffer and heated at 100 °C for 5 min. Eluted proteins were analyzed by western blotting.

### Immunofluorescence

For immunocytochemistry, cells were seeded in 24‐well plates and cultured with the indicated treatments. For imaging, cells were fixed in 4% paraformaldehyde for 10 min, permeabilized with 0.1% Triton X‐100 for 20 min, and blocked with goat serum at RT for 30 min. After overnight incubation with primary antibody (anti‐HCG, ABclonal; A27815 or anti‐E‐cadherin, Abcam; ab314063) diluted according to the manufacturer's instructions at 4 °C, the cells were incubated with secondary antibodies (YSFluor594 goat anti‐rabbit IgG, Yeason; 33112ES60 or YSFluor 488 rabbit anti‐mouse IgG, Yeason; 33906ES60) at RT for 30 min, followed by DAPI staining for 5 min. Human villous tissues were fixed with 4% paraformaldehyde, paraffin‐embedded, and sectioned into 3 µm slices. Antigen retrieval was performed on the tissue sections using Tris‐EDTA (pH 9.0), and the same immunofluorescence procedure used for the cells was followed. Anti‐BHLHE40 (Proteintech; 17895‐1‐AP) and anti‐HLA‐G (ABclonal; A24425) were used as primary antibodies.

### ELISA

Similar numbers of cells (5×10^5^) were seeded per well in six‐well plates and cultured for two days. After collecting the supernatant, cells were recounted, and data were normalized to cell counts. However, it is important to point out that cell counting might not be accurate for multinucleated cells, which is a limitation. Conditioned media were analyzed using human E2 (YOBIBIO U96‐1049E), progesterone (YOBIBIO U96‐3494E) and HCG (YOBIBIO U96‐1292E) ELISA kits according to the manufacturer's instructions. Briefly, standards of varying concentrations and supernatants were added to the wells and incubated at 37 °C for 50 min. After washing, a biotinylated antibody solution was added and incubated at 37 °C for 50 min. The wells were then incubated with SABC solution and TMB chromogenic solution at 37 °C for 30 and 20 min, respectively, with the final incubation performed in the dark. The reaction was terminated using a stop solution. Optical density (OD) values were measured at 450 nm in a microplate reader for 30 min. Sample concentrations were determined from the OD values and the standard curve.

### Statistical Analysis

The software GraphPad Prism (v8.0) was used for data analysis. All data were displayed as the mean ± standard error of the mean (SEM), with n values indicating the number of samples or independent experiments, as described in the figure legends. The data were tested for normality. Two‐tailed paired or unpaired Student's *t*‐test was used in a two‐group comparison, which was detailed in the figure legends. The Wilcoxon signed‐rank test was used in snRNA‐seq analysis. For all tests, a *p*‐value < 0.05 was considered statistically significant. Significance was shown using asterisks which were specified in legends.

## Conflict of Interest

The authors declare no conflict of interest.

## Author Contributions

L.P., W.Z., C.X., and Y.L. contributed equally to this work. L.P., W.Z., and M.D. conceived the idea, designed the experiments, and wrote the paper. L.P. and C.X. conducted the experiments. Y.L. performed the bioinformatics analyses. H.W., Q.W. and P.C. provided advice and carefully edited the manuscript. Y.L., J.G., and T.Z. contributed to the performance of the experiments and commented on the manuscript. M.D., S.G., and W.Z. supervised the project. M.D., Y.W., P.C., and Q.W. revised the manuscript. All authors approved the final manuscript.

## Supporting information



Supporting Information

Supporting Data

## Data Availability

The data that support the findings of this study are available in the supplementary material of this article.

## References

[1] I. Brosens , R. Pijnenborg , L. Vercruysse , R. Romero , Am. J. Obstet. Gynecol. 2011, 204, 193. 21094932 10.1016/j.ajog.2010.08.009 PMC3369813

[2] M. Hemberger , C. W. Hanna , W. Dean , Nat. Rev. Genet. 2020, 21, 27. 31534202 10.1038/s41576-019-0169-4

[3] S. J. Renaud , M. J. Jeyarajah , Cell. Mol. Life Sci. 2022, 79, 433. 35859055 10.1007/s00018-022-04475-z PMC11072895

[4] A. J. Wilcox , C. R. Weinberg , J. F. O'Connor , D. D. Baird , J. P. Schlatterer , R. E. Canfield , E. G. Armstrong , B. C. Nisula , N. Engl. J. Med. 1988, 319, 189. 3393170 10.1056/NEJM198807283190401

[5] L. Paulesu , C. V. Rao , F. Ietta , A. Pietropolli , C. Ticconi , Int. J. Mol. Sci. 2018, 19, 914. 29558393 10.3390/ijms19030914 PMC5877775

[6] T. Fournier , Ann. Endocrinol. (Paris) 2016, 77, 75. 27177499 10.1016/j.ando.2016.04.012

[7] V. Shankar , C. van Blitterswijk , E. Vrij , S. Giselbrecht , Adv. Sci. (Weinh) 2021, 8, 2004250. 33898195 10.1002/advs.202004250 PMC8061376

[8] H. Okae , H. Toh , T. Sato , H. Hiura , S. Takahashi , K. Shirane , Y. Kabayama , M. Suyama , H. Sasaki , T. Arima , Cell Stem Cell 2018, 22, 50. 29249463 10.1016/j.stem.2017.11.004

[9] Y. Wei , T. Wang , L. Ma , Y. Zhang , Y. Zhao , K. Lye , L. Xiao , C. Chen , Z. Wang , Y. Ma , X. Zhou , F. Sun , W. Li , C. Dunk , S. Li , A. Nagy , Y. Yu , G. Pan , S. J. Lye , Y. Shan , Sci. Adv. 2021, 7, abf4416. 10.1126/sciadv.abf4416 PMC8357231 34380613

[10] C. Yi , H. Song , H. Liang , Y. Ran , J. Tang , E. Chen , F. Li , L. Fu , Y. Wang , F. Chen , Y. Wang , Y. Ding , Y. Xie , Int. J. Biol. Macromol. 2024, 263, 130220. 38368983 10.1016/j.ijbiomac.2024.130220

[11] A. J. van Voorden , R. Keijser , G. J. M. Veenboer , S. A. Lopes Cardozo , D. Diek , J. A. Vlaardingerbroek , M. van Dijk , C. Ris‐Stalpers , A. M. M. van Pelt , G. B. Afink , Proc. Natl. Acad. Sci. USA 2023, 120, 2217405120. 10.1073/pnas.2217405120 PMC10334808 37406095

[12] C. Du , J. Jiang , Y. Li , M. Yu , J. Jin , S. Chen , H. Fan , T. S. Macfarlan , B. Cao , M.‐A. Sun , Genome Res. 2023, 33, 197. 36806146 10.1101/gr.277150.122 PMC10069462

[13] W. Zheng , Y. Zhang , P. Xu , Z. Wang , X. Shao , C. Chen , H. Cai , Y. Wang , M. Sun , W. Deng , F. Liu , J. Lu , X. Zhang , D. Cheng , I. U. Mysorekar , H. Wang , Y.‐L. Wang , X. Hu , B. Cao , Proc. Natl. Acad. Sci. USA 2024, 121, 2404062121. 10.1073/pnas.2404062121 PMC11253012 38968109

[14] M. Yu , X. Hu , Z. Pan , C. Du , J. Jiang , W. Zheng , H. Cai , Y. Wang , W. Deng , H. Wang , J. Lu , M.‐A. Sun , B. Cao , Nucleic Acids Res. 2023, 51, 4745. 36864754 10.1093/nar/gkad109 PMC10250217

[15] A. Paquette , K. Ahuna , Y. M. Hwang , J. Pearl , H. Liao , P. Shannon , L. Kadam , S. Lapehn , M. Bucher , R. Roper , C. Funk , J. MacDonald , T. Bammler , P. Baloni , H. Brockway , W. A. Mason , N. Bush , K. Z. Lewinn , C. J. Karr , J. Stamatoyannopoulos , L. J. Muglia , H. Jones , Y. Sadovsky , L. Myatt , S. Sathyanarayana , N. D. Price , Sci. Adv. 2024, 10, adf3411. 10.1126/sciadv.adf3411 PMC11212735 38941464

[16] B. Saha , A. Ganguly , P. Home , B. Bhattacharya , S. Ray , A. Ghosh , M. A. K. Rumi , C. Marsh , V. A. French , S. Gunewardena , S. Paul , Proc. Natl. Acad. Sci. USA 2020, 117, 17864. 32669432 10.1073/pnas.2002449117 PMC7395512

[17] R. Hornbachner , A. Lackner , H. Papuchova , S. Haider , M. Knöfler , K. Mechtler , P. A. Latos , Proc. Natl. Acad. Sci. USA 2021, 118, 2105130118. 10.1073/pnas.2105130118 PMC8449346 34507999

[18] T. Mizutani , M. Orisaka , Y. Miyazaki , R. Morichika , M. Uesaka , K. Miyamoto , Y. Yoshida , Mol. Hum. Reprod. 2022, 28, gaac032. 35993908 10.1093/molehr/gaac032

[19] G. Jaju Bhattad , M. J. Jeyarajah , M. G. McGill , V. Dumeaux , H. Okae , T. Arima , P. Lajoie , N. G. Bérubé , S. J. Renaud , Cell Death Dis. 2020, 11, 311. 32366868 10.1038/s41419-020-2500-6 PMC7198514

[20] L.‐J. Wang , C.‐P. Chen , Y.‐S. Lee , P.‐S. Ng , G.‐D. Chang , Y.‐H. Pao , H.‐F. Lo , C.‐H. Peng , M.‐L. Cheong , H. Chen , Nat. Commun. 2022, 13, 1626. 35338152 10.1038/s41467-022-29312-6 PMC8956607

[21] S. J. Renaud , D. Chakraborty , C. W. Mason , M. A. Rumi , J. L. Vivian , M. J. Soares , Proc. Natl. Acad. Sci. USA 2015, 112, E6175. 26504231 10.1073/pnas.1507397112 PMC4653227

[22] M. Cesana , G. Tufano , F. Panariello , N. Zampelli , C. Soldati , M. Mutarelli , S. Montefusco , G. Grieco , L. V. Sepe , B. Rossi , E. Nusco , G. Rossignoli , G. Panebianco , F. Merciai , E. Salviati , E. M. Sommella , P. Campiglia , G. Martello , D. Cacchiarelli , D. L. Medina , A. Ballabio , Cell Death Differ. 2024, 31, 1439. 38965447 10.1038/s41418-024-01337-y PMC11519894

[23] C. Krendl , D. Shaposhnikov , V. Rishko , C. Ori , C. Ziegenhain , S. Sass , L. Simon , N. S. Müller , T. Straub , K. E. Brooks , S. L. Chavez , W. Enard , F. J. Theis , M. Drukker , Proc. Natl. Acad. Sci. USA 2017, 114, E9579. 29078328 10.1073/pnas.1708341114 PMC5692555

[24] A. Ghosh , R. Kumar , R. P. Kumar , S. Ray , A. Saha , N. Roy , P. Dasgupta , C. Marsh , S. Paul , Proc. Natl. Acad. Sci. USA 2024, 121, 2310502121. 10.1073/pnas.2310502121 PMC10895349 38346193

[25] P. Home , R. P. Kumar , A. Ganguly , B. Saha , J. Milano‐Foster , B. Bhattacharya , S. Ray , S. Gunewardena , A. Paul , S. A. Camper , P. E. Fields , Development 2017, 144, 876. 28232602 10.1242/dev.145318 PMC5374352

[26] S. Paul , P. Home , B. Bhattacharya , S. Ray , Placenta 2017, 60, S61. 28526138 10.1016/j.placenta.2017.05.005 PMC7021224

[27] M. Wang , Y. Liu , R. Sun , F. Liu , J. Li , L. Yan , J. Zhang , X. Xie , D. Li , Y. Wang , S. Li , X. Zhu , R. Li , F. Lu , Z. Xiao , H. Wang , Nat. Genet. 2024, 56, 294. 38267607 10.1038/s41588-023-01647-w PMC10864176

[28] G. Meinhardt , P. Husslein , M. Knöfler , Placenta 2005, 26, 527. 15993702 10.1016/j.placenta.2004.09.005

[29] R. Hu , Q. Wang , Y. Jia , Y. Zhang , B. Wu , S. Tian , Y. Wang , Y. Wang , W. Ma , Tissue Cell 2021, 73, 101616. 34481230 10.1016/j.tice.2021.101616

[30] D. Lei , T. Chen , C. Fan , Q. Xie , J. Hazard. Mater. 2024, 479, 135594. 39191013 10.1016/j.jhazmat.2024.135594

[31] R. N. Pillai , J. C. Konje , D. G. Tincello , N. Potdar , Hum. Reprod. Update 2016, 22, 228. 26663220 10.1093/humupd/dmv054

[32] J. Johns , S. Muttukrishna , M. Lygnos , N. Groome , E. Jauniaux , Reprod. Biomed Online 2007, 15, 413. 17908404 10.1016/s1472-6483(10)60367-7

[33] A. Freis , J. Schlegel , V. Daniel , J. Jauckus , T. Strowitzki , A. Germeyer , Reprod. Biol. Endocrinol. 2018, 16, 93. 30266090 10.1186/s12958-018-0411-5 PMC6162891

[34] P. Sirikunalai , C. Wanapirak , S. Sirichotiyakul , F. Tongprasert , K. Srisupundit , S. Luewan , K. Traisrisilp , T. Tongsong , J. Obstet. Gynaecol. 2016, 36, 178. 26368010 10.3109/01443615.2015.1036400

[35] Y. Fu , Y. Song , J. Zhang , L. P. Wei , X. R. Sun , J. Reprod. Immunol. 2023, 155, 103784. 36508844 10.1016/j.jri.2022.103784

[36] P. Gerbaud , K. Taskén , G. Pidoux , Front. Pharmacol. 2015, 6, 202. 26441659 10.3389/fphar.2015.00202 PMC4569887

[37] H. Zhou , C. Zhao , P. Wang , W. Yang , H. Zhu , S. Zhang , Front. Endocrinol. (Lausanne) 2023, 14, 1107182. 36798658 10.3389/fendo.2023.1107182 PMC9927020

[38] M. Shen , T. Kawamoto , M. Teramoto , S. Makihira , K. Fujimoto , W. Yan , M. Noshiro , Y. Kato , Eur. J. Cell Biol. 2001, 80, 329. 11432722 10.1078/0171-9335-00167

[39] K. Miyazaki , T. Kawamoto , K. Tanimoto , M. Nishiyama , H. Honda , Y. Kato , J. Biol. Chem. 2002, 277, 47014. 12354771 10.1074/jbc.M204938200

[40] B. Zhang , H. Zheng , B. Huang , W. Li , Y. Xiang , X. Peng , J. Ming , X. Wu , Y. Zhang , Q. Xu , W. Liu , X. Kou , Y. Zhao , W. He , C. Li , B. Chen , Y. Li , Q. Wang , J. Ma , Q. Yin , K. Kee , A. Meng , S. Gao , F. Xu , J. Na , W. Xie , Nature 2016, 537, 553. 27626382 10.1038/nature19361

[41] K. Wu , D. Fan , H. Zhao , Z. Liu , Z. Hou , W. Tao , G. Yu , S. Yuan , X. Zhu , M. Kang , Y. Tian , Z.‐J. Chen , J. Liu , L. Gao , Cell Discov. 2023, 9, 29. 36914622 10.1038/s41421-022-00514-y PMC10011383

[42] A. C. H. Chen , Y. L. Lee , H. Ruan , W. Huang , S. W. Fong , S. Tian , K. C. Lee , G. M. Wu , Y. Tan , T. C. H. Wong , J. Wu , W. Zhang , D. Cao , J. F. C. Chow , P. Liu , W. S. B. Yeung , Adv. Sci. (Weinh) 2023, 10, 2204797. 36775869 10.1002/advs.202204797 PMC10104645

[43] F. Chi , M. S. Sharpley , R. Nagaraj , S. S. Roy , U. Banerjee , Dev. Cell 2020, 53, 24. 10.1016/j.devcel.2020.02.015 PMC7289320 32197068

[44] L. B. Chopp , X. Zhu , Y. Gao , J. Nie , J. Singh , P. Kumar , K. Z. Young , S. Patel , C. Li , M. Balmaceno‐Criss , M. S. Vacchio , M. M. Wang , F. Livak , J. L. Merchant , L. Wang , M. C. Kelly , J. Zhu , R. Bosselut , Sci. Immunol. 2023, 8, adi9066. 10.1126/sciimmunol.adi9066 PMC12291171 37948511

[45] W. D. Gifford , S. L. Pfaff , T. S. Macfarlan , Trends Cell Biol. 2013, 23, 218. 23411159 10.1016/j.tcb.2013.01.001 PMC4034679

[46] E. B. Chuong , BioEssays 2013, 35, 853. 23873343 10.1002/bies.201300059 PMC4332834

[47] M. Knöfler , S. Haider , L. Saleh , J. Pollheimer , T. Gamage , J. James , Cell. Mol. Life Sci. 2019, 76, 3479. 31049600 10.1007/s00018-019-03104-6 PMC6697717

[48] C. Mi , B. Ye , Z. Gao , J. Du , R. Li , D. Huang , Mol. Hum. Reprod. 2020, 26, 532. 32579212 10.1093/molehr/gaaa037

[49] H. Sun , R. Taneja , Proc. Natl. Acad. Sci. USA 2000, 97, 4058. 10737769 10.1073/pnas.070526297 PMC18147

[50] M. Kanda , H. Yamanaka , S. Kojo , Y. Usui , H. Honda , Y. Sotomaru , M. Harada , M. Taniguchi , N. Suzuki , T. Atsumi , H. Wada , M. Baghdadi , K.‐I. Seino , Proc. Natl. Acad. Sci. USA 2016, 113, E3394. 27226296 10.1073/pnas.1604178113 PMC4914147

[51] K. Miyazaki , M. Miyazaki , Y. Guo , N. Yamasaki , M. Kanno , Z.‐I. Honda , H. Oda , H. Kawamoto , H. Honda , J. Immunol. 2010, 185, 7330. 21057086 10.4049/jimmunol.1001381

[52] M. E. Cook , N. N. Jarjour , C. C. Lin , B. T. Edelson , Trends Immunol. 2020, 41, 1023. 33039338 10.1016/j.it.2020.09.002 PMC7606821

[53] F. Sato , U. K. Bhawal , T. Yoshimura , Y. Muragaki , J. Cancer 2016, 7, 153. 26819638 10.7150/jca.13748 PMC4716847

[54] Z. Yun , H. L. Maecker , R. S. Johnson , A. J. Giaccia , Dev. Cell 2002, 2, 331. 11879638 10.1016/s1534-5807(02)00131-4

[55] Y. Cao , X. Wang , Y. Liu , P. Liu , J. Qin , Y. Zhu , S. Zhai , Y. Jiang , Y. Liu , L. Han , J. Luo , R. Zhang , M. Shi , L. Wang , X. Tang , M. Xue , J. Liu , W. Wang , C. Wen , X. Deng , C. Peng , H. Chen , D. Cheng , L. Jiang , B. Shen , Adv. Sci. (Weinh) 2024, 11, 2306298. 38064101 10.1002/advs.202306298 PMC10870036

[56] L. Lunghi , E. Frigato , M. E. Ferretti , C. Biondi , C. Bertolucci , Hum. Cell 2011, 24, 161. 22038066 10.1007/s13577-011-0032-1

[57] T. Shimizu , A. Oike , E. H. Kobayashi , A. Sekiya , N. Kobayashi , S. Shibata , H. Hamada , M. Saito , N. Yaegashi , M. Suyama , T. Arima , H. Okae , Proc. Natl. Acad. Sci. USA 2023, 120, 2311372120. 10.1073/pnas.2311372120 PMC10742386 38085778

[58] M. Hughes , N. Dobric , I. C. Scott , L. Su , M. Starovic , B. St‐Pierre , S. E. Egan , J. C. P. Kingdom , J. C. Cross , Dev Biol. 2004, 271, 26. 15196947 10.1016/j.ydbio.2004.03.029

[59] A. H. El‐Hashash , P. Esbrit , S. J. Kimber , Differentiation 2005, 73, 154. 15901283 10.1111/j.1432-0436.2005.00013.x

[60] H. Sun , B. Lu , R. Q. Li , R. A. Flavell , R. Taneja , Nat. Immunol. 2001, 2, 1040. 11668339 10.1038/ni721

[61] F. Varzideh , J. Gambardella , U. Kansakar , S. S. Jankauskas , G. Santulli , Int. J. Mol. Sci. 2023, 24, 8386. 37176093 10.3390/ijms24098386 PMC10179698

[62] M. Völker‐Albert , A. Bronkhorst , S. Holdenrieder , A. Imhof , Stem Cell Rep. 2020, 15, 1196. 10.1016/j.stemcr.2020.11.002 PMC7724464 33296672

[63] C.‐Q. E. Lee , L. Gardner , M. Turco , N. Zhao , M.‐J. Murray , N. Coleman , J. Rossant , M. Hemberger , A. Moffett , Stem Cell Rep. 2016, 6, 257. 10.1016/j.stemcr.2016.01.006 PMC4750161 26862703

[64] L. M. R. Ferreira , T. B. Meissner , T. S. Mikkelsen , W. Mallard , C. W. O'Donnell , T. Tilburgs , H. A. B. Gomes , R. Camahort , R. I. Sherwood , D. K. Gifford , J. L. Rinn , C. A. Cowan , J. L. Strominger , Proc. Natl. Acad. Sci. USA 2016, 113, 5364. 27078102 10.1073/pnas.1602886113 PMC4868469

[65] J. Boyes , P. Byfield , Y. Nakatani , V. Ogryzko , Nature 1998, 396, 594. 9859997 10.1038/25166

[66] B. Yan , J. Yang , M. Y. Kim , H. Luo , N. Cesari , T. Yang , J. Strouboulis , J. Zhang , R. Hardison , S. Huang , Y. Qiu , Nucleic Acids Res. 2021, 49, 9783. 34450641 10.1093/nar/gkab737 PMC8464053

[67] S. P. Hsiao , K. M. Huang , H. Y. Chang , S. L. Chen , Biochem. J. 2009, 422, 343. 19522704 10.1042/BJ20090072

[68] Y. Y. Wan , Trends Immunol. 2014, 35, 233. 24786134 10.1016/j.it.2014.04.002 PMC4045638

[69] W. Zheng , R. A. Flavell , Cell 1997, 89, 587.9160750

[70] Y. Wang , M. A. Su , Y. Y. Wan , Immunity 2011, 35, 337. 21924928 10.1016/j.immuni.2011.08.012 PMC3182399

[71] C.‐C. Lin , T. R. Bradstreet , E. A. Schwarzkopf , J. Sim , J. A. Carrero , C. Chou , L. E. Cook , T. Egawa , R. Taneja , T. L. Murphy , J. H. Russell , B. T. Edelson , Nat. Commun. 2014, 5, 3551. 24699451 10.1038/ncomms4551 PMC4016562

[72] F. Yu , S. Sharma , D. Jankovic , R. K. Gurram , P. Su , G. Hu , R. Li , S. Rieder , K. Zhao , B. Sun , J. Zhu , J. Exp. Med. 2018, 215, 1813. 29773643 10.1084/jem.20170155 PMC6028509

[73] M. J. Butcher , R. K. Gurram , X. Zhu , X. Chen , G. Hu , V. Lazarevic , K. Zhao , J. Zhu , Front Immunol. 2023, 14, 1186580. 37449212 10.3389/fimmu.2023.1186580 PMC10337884

[74] D. Aran , A. P. Looney , L. Liu , E. Wu , V. Fong , A. Hsu , S. Chak , R. P. Naikawadi , P. J. Wolters , A. R. Abate , A. J. Butte , M. Bhattacharya , Nat. Immunol. 2019, 20, 163. 30643263 10.1038/s41590-018-0276-y PMC6340744

[75] S. Aibar , C. B. González‐Blas , T. Moerman , V. A. Huynh‐Thu , H. Imrichova , G. Hulselmans , F. Rambow , J.‐C. Marine , P. Geurts , J. Aerts , J. van den Oord , Z. K. Atak , J. Wouters , S. Aerts , Nat. Methods 2017, 14, 1083. 28991892 10.1038/nmeth.4463 PMC5937676

[76] H. S. Kaya‐Okur , S. J. Wu , C. A. Codomo , E. S. Pledger , T. D. Bryson , J. G. Henikoff , K. Ahmad , S. Henikoff , Nat. Commun. 2019, 10, 1930. 31036827 10.1038/s41467-019-09982-5 PMC6488672

[77] Y. Zhang , T. Liu , C. A. Meyer , J. Eeckhoute , D. S. Johnson , B. E. Bernstein , C. Nusbaum , R. M. Myers , M. Brown , W. Li , X. S. Liu , Genome Biol. 2008, 9, R137. 18798982 10.1186/gb-2008-9-9-r137 PMC2592715

[78] C. S. Ross‐Innes , R. Stark , A. E. Teschendorff , K. A. Holmes , H. R. Ali , M. J. Dunning , G. D. Brown , O. Gojis , I. O. Ellis , A. R. Green , S. Ali , S.‐F. Chin , C. Palmieri , C. Caldas , J. S. Carroll , Nature 2012, 481, 389. 22217937 10.1038/nature10730 PMC3272464

[79] A. R. Quinlan , I. M. Hall , Bioinformatics 2010, 26, 841. 20110278 10.1093/bioinformatics/btq033 PMC2832824

