# Supplementary material for: BHLHE40 Cooperates with GATA2/3 to Control Human Syncytiotrophoblast Lineage Differentiation
Source: Adv Sci (Weinh). 2025 Sep 5;12(44):e07642. doi: 10.1002/advs.202507642 (PMC12667488; doi:10.1002/advs.202507642)
Supplement: Supplementary file 1 — Supporting Information [file ADVS-12-e07642-s001.docx]

((Supporting Information can be included here using this template))

Supporting Information

BHLHE40 cooperates with GATA2/3 to control human syncytiotrophoblast lineage differentiation

Lijin Peng, Weijie Zhao, Chunfang Xu, Yue Li, Jiani Guo, Taotao Zhou, Philip Chiu, Huimei Wu, Qingyu Wu, Yanxing Wei*, Shaorong Gao*, Meirong Du*


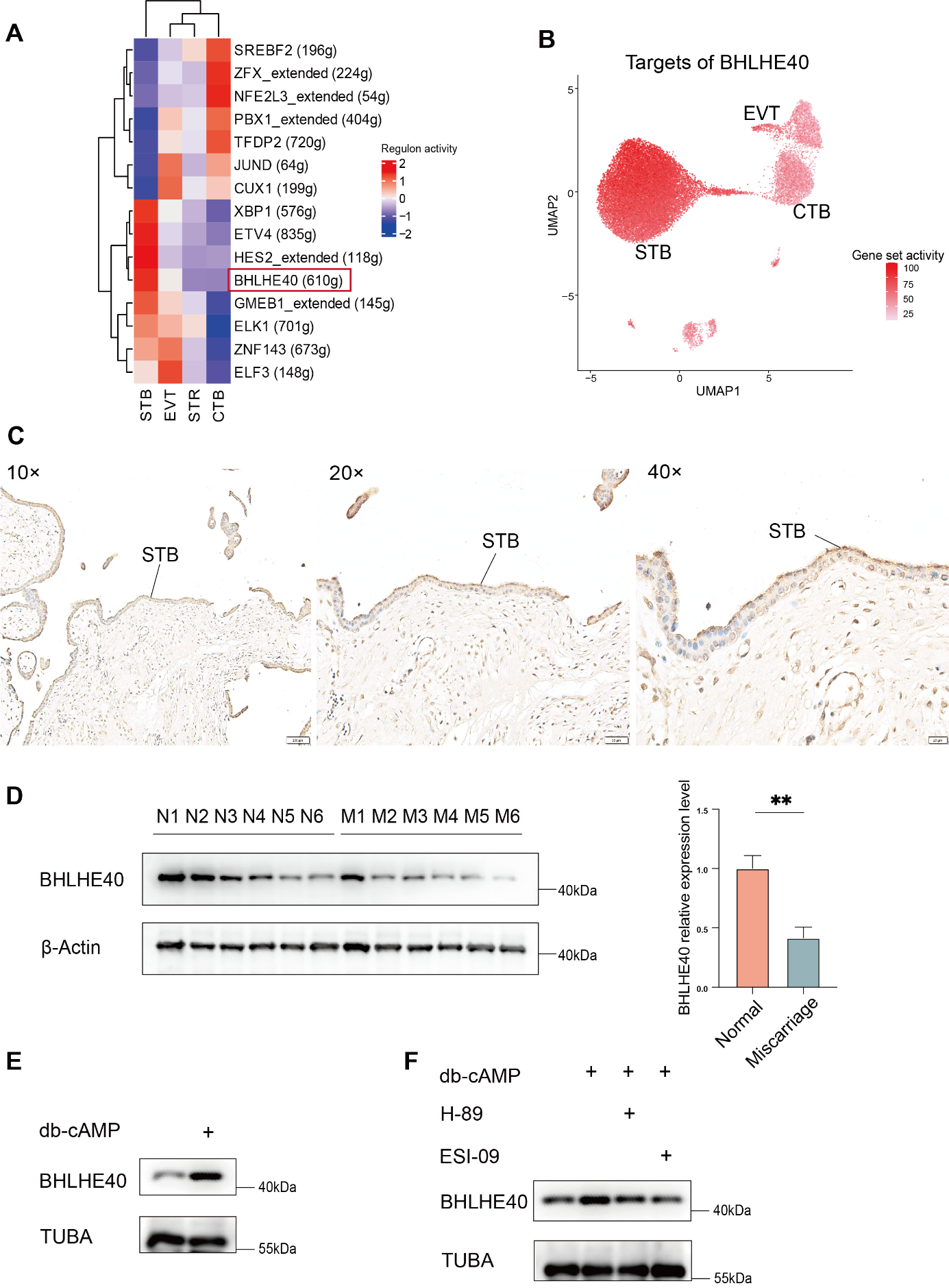


**Figure S1.** Potential regulatory role of BHLHE40 in STBs. A) Heatmap of the area under the curve (AUC) scores of TFs predicted per cell by SCENIC, showing regulatory activity of the top activated motifs in CTBs and STBs. B) SCENIC analysis predicted target genes of BHLHE40, displaying an enrichment in STBs. C) Immunohistochemistry images showing the expression of BHLHE40 in human primary villous samples obtained at 8 weeks of gestation. D) Western blotting of BHLHE40 in human primary villous samples obtained at 8 weeks of gestation (left). N: normal pregnancy; M: miscarriage. Densitometry analysis of protein expression levels was quantified using ImageJ (right). Data presented as means ± SEM, n=6, ***p* < 0.01. *P*-values were calculated using two-tailed unpaired Student's t-test. β-Actin was used as a loading control. E) Western blotting of BHLHE40 in TSCs treated by 1 μM db-cAMP or vehicle control for 48 h. Tubulin (TUBA) was used as a loading control. F) Western blotting of BHLHE40 in TSCs treated by 1 μM db-cAMP and H-89 (8 μM) or ESI-09 (8 μM) for 48 h. Tubulin (TUBA) was used as a loading control.


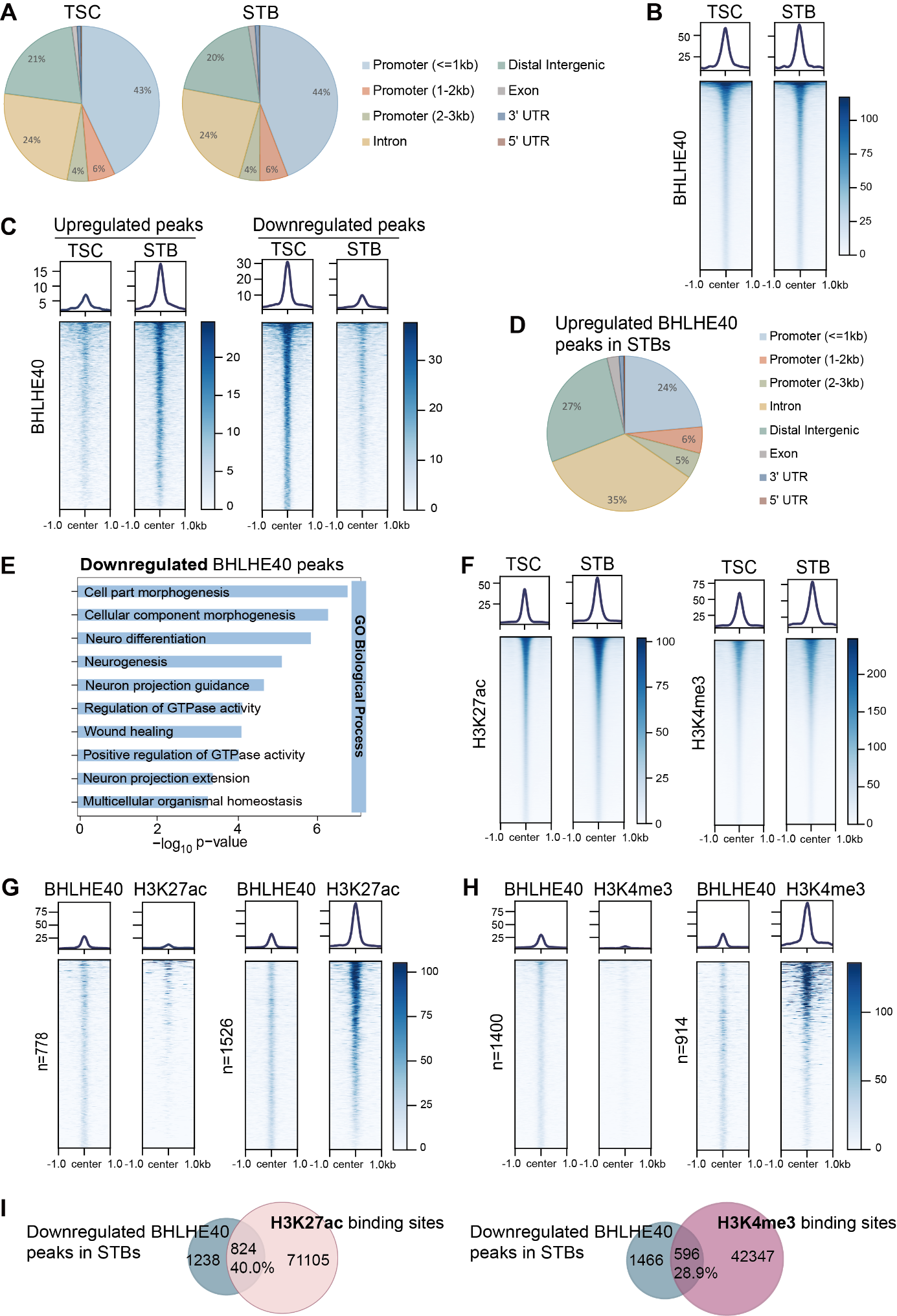


**Figure S2.** Genome-wide profiling of BHLHE40 binding loci in STBs. A) Genomic distribution of BHLHE40 binding sites in TSCs and STBs. B) Profile plot and heatmap showing BHLHE40 signals in TSCs and STBs. C) Profile plot and heatmap showing BHLHE40 signals at upregulated (red sites in Fig. 2A) and downregulated sites (blue sites in Fig. 2A) in STBs versus TSCs. D) Genomic distribution of upregulated BHLHE40 peaks in STBs. E) Selected GO terms enriched for downregulated BHLHE40 peaks in STBs compared to those in TSCs. F) Profile plot and heatmap showing H3K27ac (left) and H3K4me3 (right) signals in TSCs and STBs. G) Profile plot and heatmap showing BHLHE40 and H3K27ac signals at upregulated BHLHE40 unique peaks and overlapped peaks with H3K27ac. H) Profile plot and heatmap showing BHLHE40 and H3K4me3 signals at upregulated BHLHE40 unique peaks and overlapped peaks with H3K4me3. I) Venn diagram showing the overlap between downregulated BHLHE40 peaks and H3K27ac or H3K4me3 binding sites in STBs.


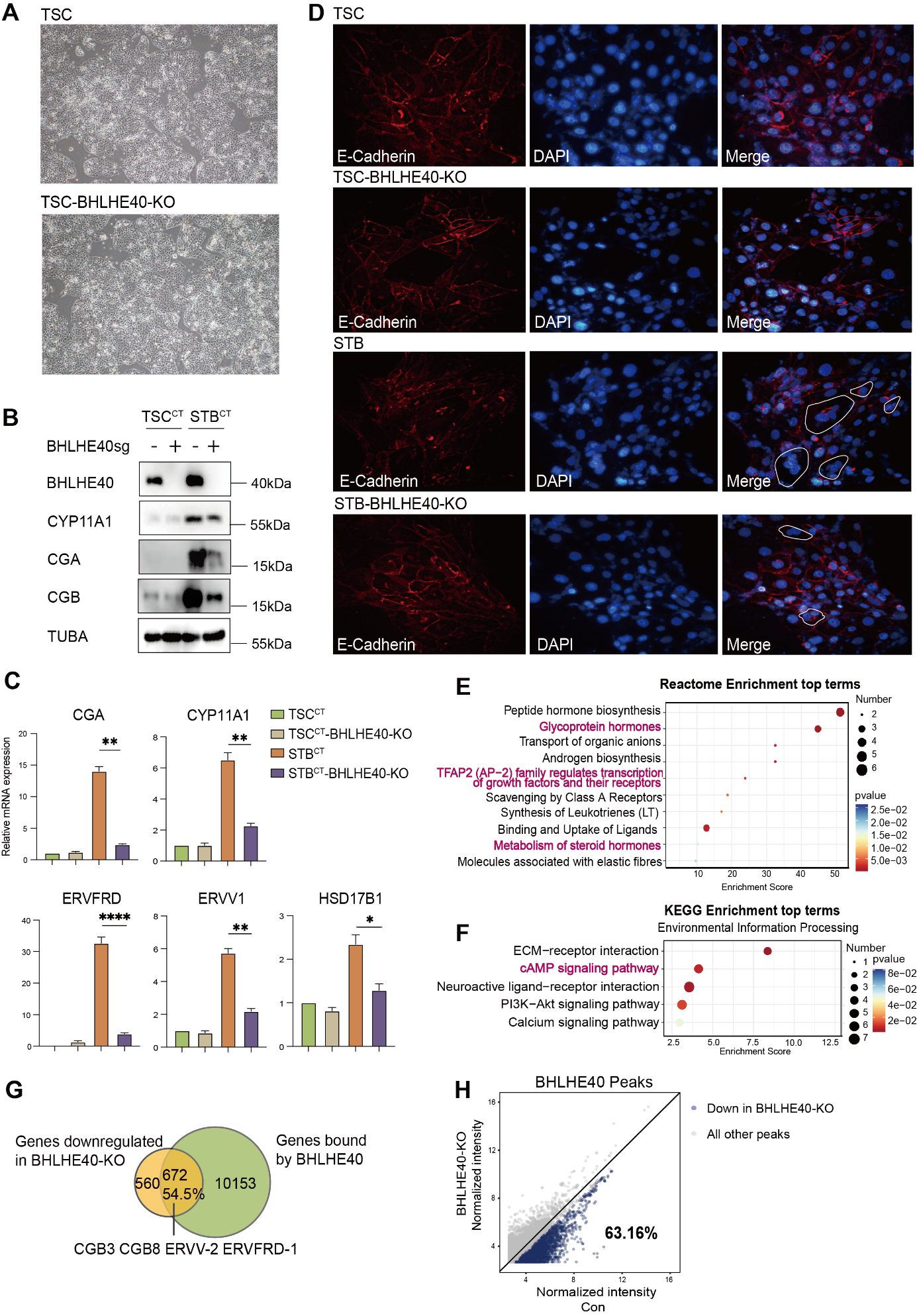


**Figure S3.** Depletion of *BHLHE40* impaired STB differentiation. A) Images of normal and *BHLHE40*-KO TSCs, Scale bar: 40 µm. B) Western blotting of BHLHE40 and STB markers in wild-type (WT) and *BHLHE40*-KO TSCs^CT^ and STBs^CT^. Tubulin (TUBA) was a loading control. C) Relative mRNA expression of STB markers in WT and *BHLHE40*-KO TSCs^CT^ and STBs^CT^. Data presented as means ± SEM, n=3, **p* < 0.05, ***p* < 0.01, *****p* < 0.0001. *P*-values were calculated using two-tailed paired Student's t-test. D) Immunofluorescence staining of E-cadherin (red) in WT and *BHLHE40*-KO TSCs and STBs. DAPI staining indicates nuclei (blue). E) Top 10 enriched Reactome terms for decreased genes in *BHLHE40*-KO STBs compared to those in WT STBs. F) Top enriched KEGG pathways for decreased genes in *BHLHE40*-KO STBs compared those in WT STBs. G) Venn diagram showing the overlap between downregulated genes in *BHLHE40*-KO STBs compared to the WT and BHLHE40 binding genes identified in STBs. H) Differential BHLHE40 CUT&Tag-seq analysis comparing *BHLHE40*-KO versus WT STBs. The sites with lower signals in *BHLHE40*-KO were in blue and others were in gray. Among them, 63.16% of BHLHE40 peaks were reduced in *BHLHE40*-KO versus WT STBs. *p*-adj < 0.05.


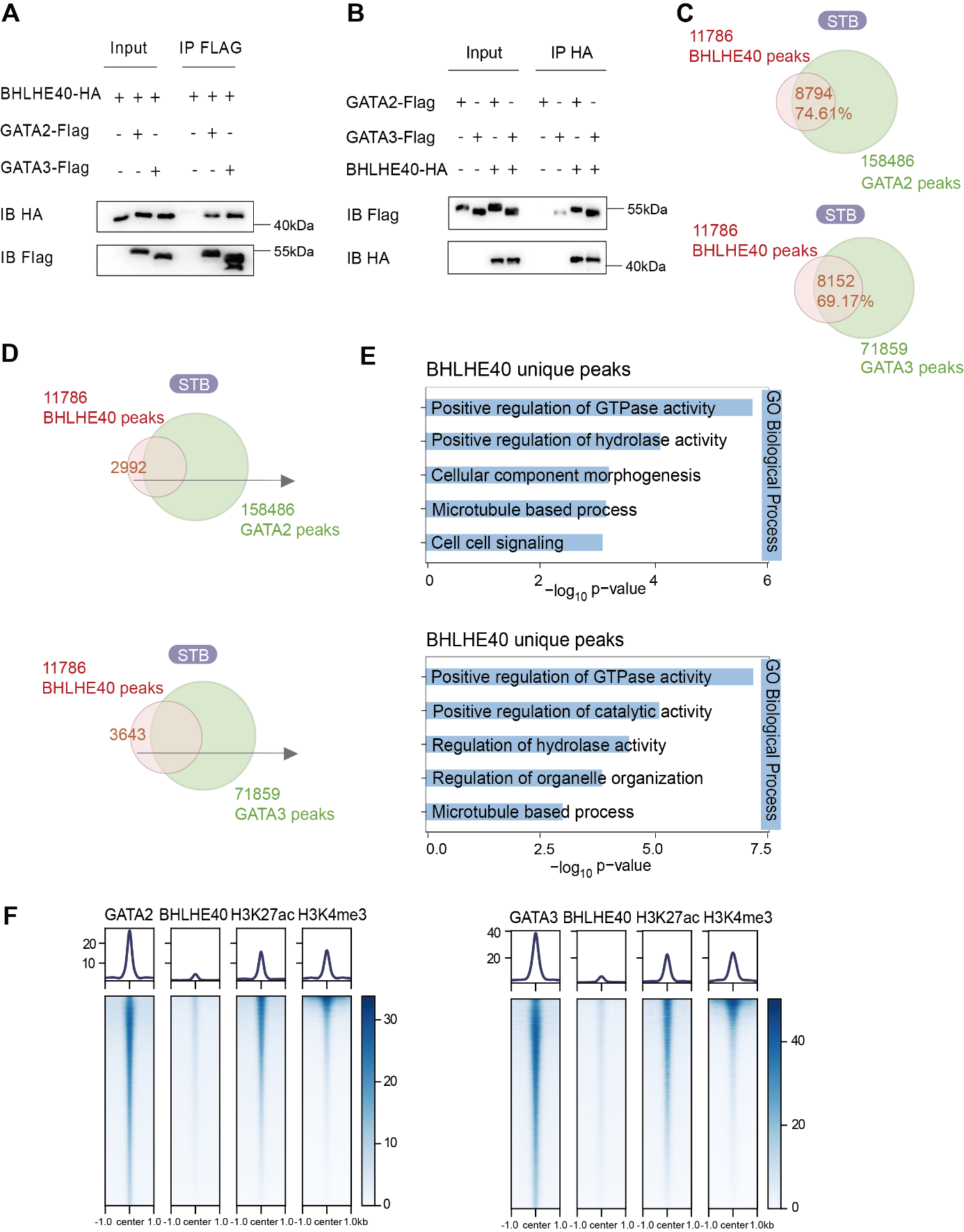


**Figure S4.** BHLHE40 interacts with GATA2/3. A) HEK293T cells were transfected with FLAG-tagged GATA2/3 and HA-tagged BHLHE40. FLAG was pulled down and eluted proteins were analyzed by western blotting. B) HEK293T cells were transfected with FLAG-tagged GATA2/3 and HA-tagged BHLHE40. HA was pulled down and eluted proteins were analyzed by western blotting. C) Venn diagram showing the overlap between BHLHE40 and GATA2 (up) or GATA3 (bottom) peaks in STBs. D) Venn diagram showing the BHLHE40 peaks not overlapped with GATA2 (up) or GATA3 (bottom) in STBs. E) Selected GO terms enriched for BHLHE40 peaks not overlapped with GATA2 (up) or GATA3 (bottom) in STBs. F) Profile plot and heatmap showing BHLHE40, GATA2, H3K27ac and H3K4me3 signals in GATA2 unique (left) and GATA3 unique (right) peaks not overlapping with BHLHE40.


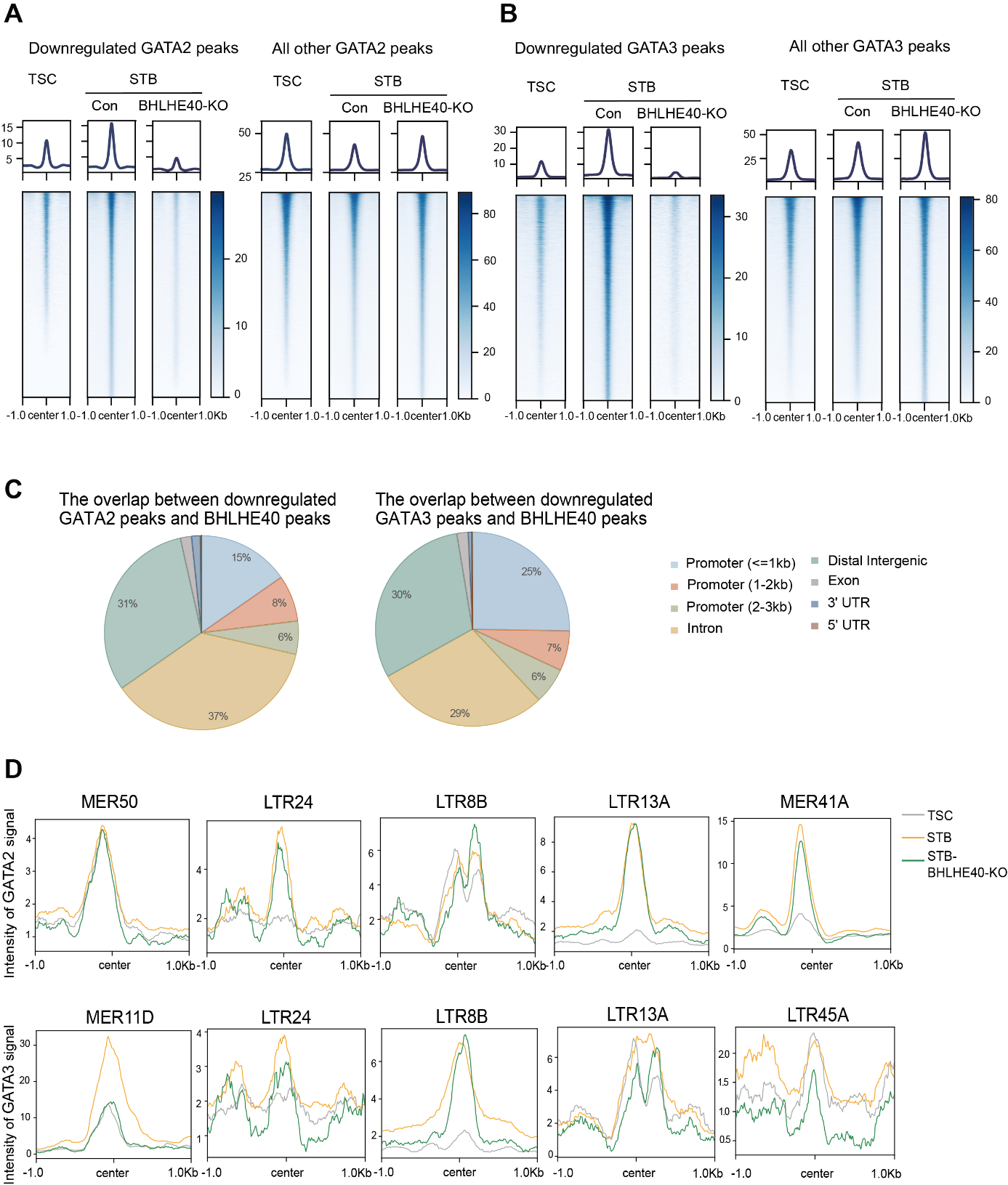


**Figure S5.** *BHLHE40*-KO alters GATA2/3 binding patterns in STBs. A) and B) Profile plots and heatmaps showing GATA2/3 signals at downregulated or all other GATA2/3 peaks in TSCs, STBs, and *BHLHE40*-KO STBs. C) Genomic distribution of overlapped peaks in Figure 5B (overlapped GATA2 (left)/GATA3 (right) loss peaks in *BHLHE40*-KO and BHLHE40 peaks in STBs). D) Profiles of GATA2/3 signal at ERV subtypes associated with STB genes in WT and *BHLHE40*-KO STBs.

**Table S1.** List of TFs identified by CGB promoter pull-down assay

| **TFs** | **FDR** | **Accession** | **Coverage** | **Area** |
| --- | --- | --- | --- | --- |
| **CTCF** | High | P49711 | 3.43879 | 9.8e+07 |
| **TERF2** | High | Q15554 | 9.778598 | 9.3e+07 |
| **RXRA** | High | P19793 | 6.709957 | 8.8e+07 |
| **YBX1** | High | P67809 | 33.02469 | 8.5e+07 |
| **ARID3A** | High | Q99856 | 9.780776 | 8.5e+07 |
| **SAFB** | High | Q15424 | 2.513661 | 8.3e+07 |
| **PURA** | High | Q00577 | 24.84472 | 7.9e+07 |
| **NFYC** | Medium | Q13952 | 1.746725 | 7.8e+07 |
| **GATAD2A** | High | Q86YP4 | 3.00158 | 7.3e+07 |
| **BHLHE40** | High | O14503 | 2.912621 | 7.2e+07 |
| **MBD2** | High | Q9UBB5 | 3.406326 | 7.1e+07 |
| **E2F3** | High | O00716 | 2.365591 | 6.9e+07 |
| **ZBTB7A** | High | O95365 | 3.938356 | 6.8e+07 |
| **DNTTIP1** | High | Q9H147 | 3.647416 | 6.5e+07 |
| **KDM2B** | High | Q8NHM5 | 0.898204 | 6.4e+07 |
| **PURB** | High | Q96QR8 | 30.12821 | 6.1e+07 |
| **KDM2A** | High | Q9Y2K7 | 2.581756 | 5.9e+07 |
| **ETV6** | Medium | P41212 | 1.99115 | 5.7e+07 |
| **HIC2** | High | Q96JB3 | 5.365854 | 5.6e+07 |
| **JUND** | High | P17535 | 4.034582 | 5.5e+07 |
| **TP63** | High | Q9H3D4 | 7.058824 | 5.4e+07 |
| **RFX5** | High | P48382 | 3.409091 | 5.4e+07 |
| **MAFF** | High | Q9ULX9 | 9.756098 | 5.4e+07 |
| **TCF7L2** | High | Q9NQB0 | 2.100162 | 5.2e+07 |
| **KLF13** | Medium | Q9Y2Y9 | 5.902778 | 5.1e+07 |
| **YY1** | High | P25490 | 3.623188 | 5e+07 |
| **TFAP2A** | High | P05549 | 6.407323 | 4.7e+07 |
| **NR2C2** | High | P49116 | 2.516779 | 4.6e+07 |
| **TBPL2** | Medium | Q6SJ96 | 1.866667 | 4.6e+07 |
| **HMG20A** | High | Q9NP66 | 5.763689 | 4.6e+07 |
| **GLI3** | High | P10071 | 0.886076 | 4.5e+07 |
| **CEBPZ** | Medium | Q03701 | 0.664137 | 4.5e+07 |
| **NKRF** | High | O15226 | 1.304348 | 3.9e+07 |
| **TIGD2** | High | Q4W5G0 | 4.380952 | 3.7e+07 |
| **CUX1** | High | P39880 | 1.92691 | 3.3e+07 |
| **ELF2** | High | Q15723 | 2.529511 | 3.2e+07 |
| **DNMT1** | High | P26358 | 1.918317 | 3e+07 |
| **L3MBTL3** | High | Q96JM7 | 1.282051 | 3e+07 |
| **RBPJ** | High | Q06330 | 2.2 | 2.8e+07 |
| **ZNF888** | High | P0CJ79 | 11.28134 | 2.8e+07 |
| **FOXK1** | High | P85037 | 3.13779 | 2.6e+07 |
| **ZNF787** | High | Q6DD87 | 3.403141 | 2.6e+07 |
| **LIN28B** | High | Q6ZN17 | 29.6 | 2.4e+07 |
| **ATF7** | High | P17544 | 5.060729 | 2.4e+07 |
| **TEAD4** | High | Q15561 | 2.304147 | 2.4e+07 |
| **ZNF845** | High | Q96IR2 | 16.49485 | 2.2e+07 |
| **ZBTB10** | High | Q96DT7 | 0.918485 | 2.2e+07 |
| **NR2F6** | High | P10588 | 16.83168 | 2.1e+07 |
| **LTF** | High | P02788 | 10.84507 | 2e+07 |
| **GLYR1** | High | Q49A26 | 14.46655 | 2e+07 |
| **NRF1** | High | Q16656 | 3.976143 | 2e+07 |
| **GZF1** | High | Q9H116 | 3.094233 | 2e+07 |
| **ZFP64** | High | Q9NPA5 | 1.468429 | 2e+07 |
| **GABPA** | High | Q06546 | 2.643172 | 2e+07 |
| **ZFAT** | Medium | Q9P243 | 0.643604 | 1.9e+07 |
| **ZNF768** | High | Q9H5H4 | 7.222222 | 1.8e+07 |
| **TSHZ3** | Medium | Q63HK5 | 0.832562 | 1.7e+07 |
| **ELF1** | High | P32519 | 5.169628 | 1.6e+07 |
| **RFX1** | High | P22670 | 1.225741 | 1.6e+07 |
| **ESRRA** | High | P11474 | 15.13002 | 1.5e+07 |
| **TBX3** | Medium | O15119 | 1.480485 | 1.5e+07 |
| **ZNF331** | High | Q9NQX6 | 4.103672 | 1.5e+07 |
| **ZNF24** | High | P17028 | 2.445652 | 1.5e+07 |
| **ZBTB7B** | High | O15156 | 4.823748 | 1.4e+07 |
| **CREB1** | High | P16220 | 2.639296 | 1.3e+07 |
| **RXRB** | High | P28702 | 5.816135 | 1.2e+07 |
| **ARID3B** | High | Q8IVW6 | 1.426025 | 1.1e+07 |
| **NR2F2** | High | P24468 | 16.18357 | 1.1e+07 |
| **ZNF687**  **ZNF143** | High  High | Q8N1G0  P52747 | 1.859337  1.253918 | 1e+07  1e+07 |

High: FDR<0.01, Medium: 0.01≤FDR<0.05

**Table S2.** List of primers used in RT-qPCR

| Gene | Forward sequence | Reverse sequence |
| --- | --- | --- |
| BHLHE40 | CAAGAGTCCGAAGAACCCCC | TGAGGCCTGGGTATAGCACT |
| CGA | TGTGCAGGATTGCCCAGAAT | ACAAGTACTGCAGTGGCACG |
| CGB | GCTGGACCAGTGAGAGGAGA | GGTAGTTGCACACCACCTGA |
| CYP11A1 | ACATCAAGGCCAACGTCACA | TAGCATCGTGGCCATGTCTC |
| CYP19A1 | TGCGAGTCTGGATCTCTGGA | CCCAAGTTTGCTGCCGAATC |
| HSD17B1 | TGGACGTGCTGGTGTGTAAC | GCTGGCGCAATAAACGTCAT |
| ERVV1 | TGATGGCCTCCTTGGAAACG | CAGGTGTAGCCAGTGTAGCC |
| ERVV2 | CAGAACGCTTCTGAATGGCA | AATGAGCCAGCTGCTAGTGC |
| ERVFRD  CGB Promoter | CAAATGGTGCAGTGACTCGG  GGCCAGGGGACCTTGAGAAC | ACAGCTTCACTTGGGTGTGA  CCTTGGTGCGTCCCCTGCCT |
