# Supplementary figures and images for: BHLHE40 Cooperates with GATA2/3 to Control Human Syncytiotrophoblast Lineage Differentiation
Source: Adv Sci (Weinh). 2025 Sep 5;12(44):e07642. doi: 10.1002/advs.202507642 (PMC12667488; doi:10.1002/advs.202507642)

**Figure 1F**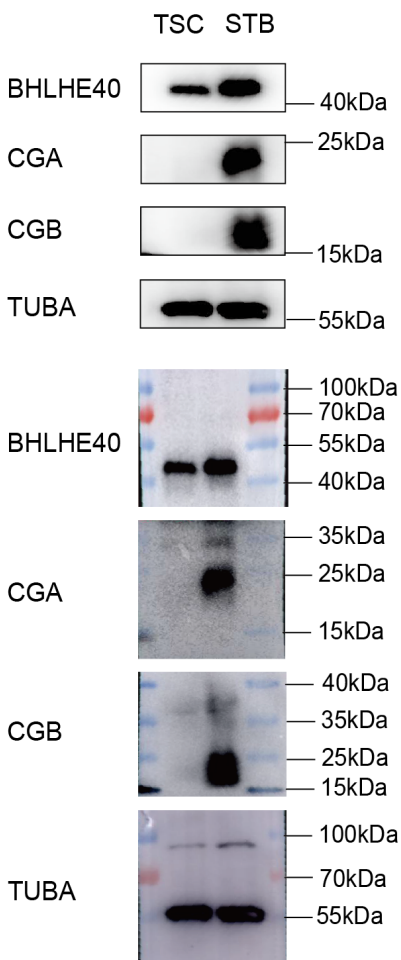**Figure 1G**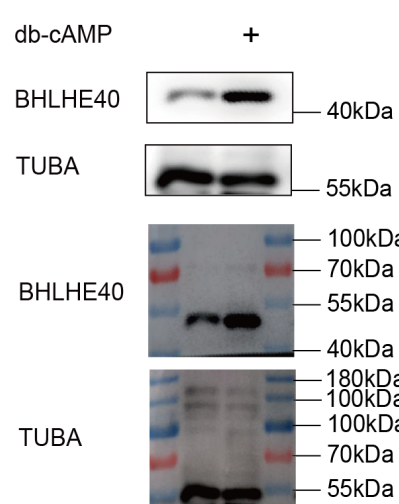**Figure 1H**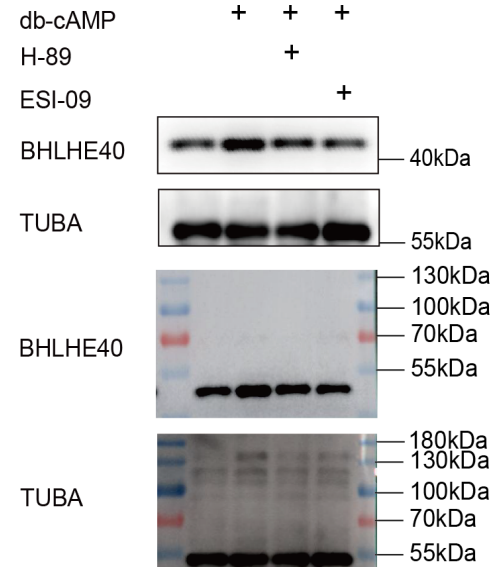**Figure S1D**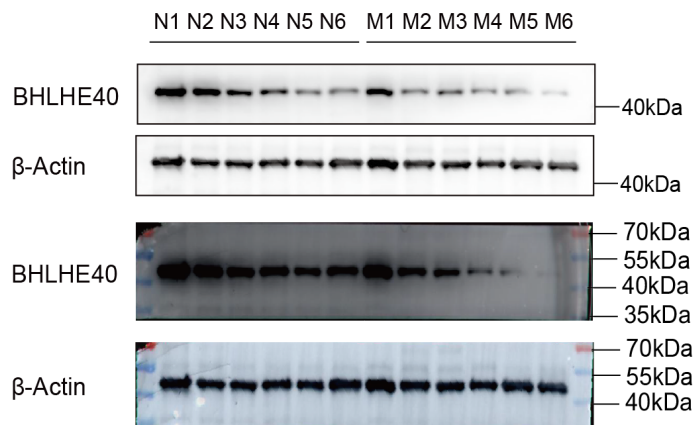**Figure 3A**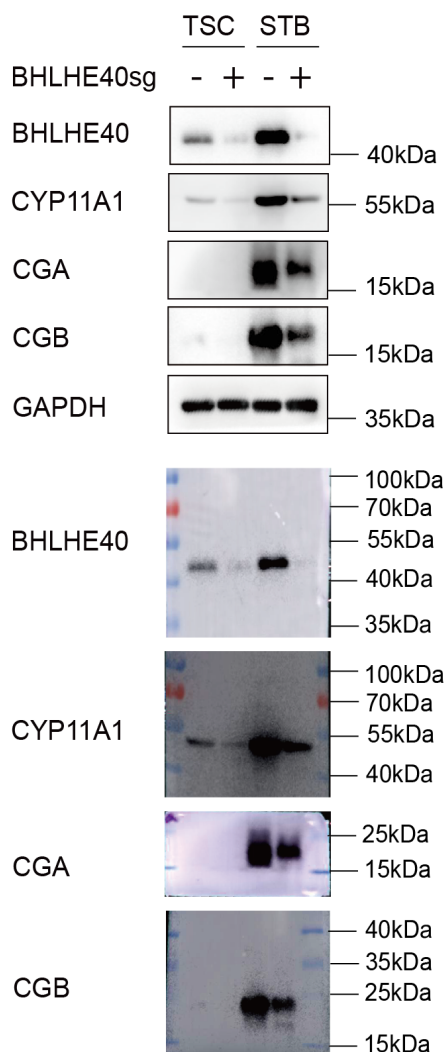**Figure S3B**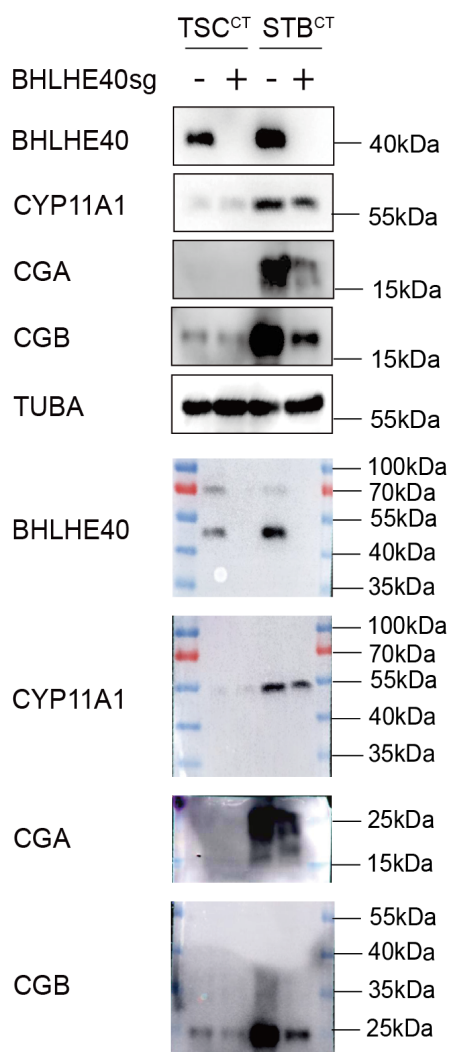**Figure 4B**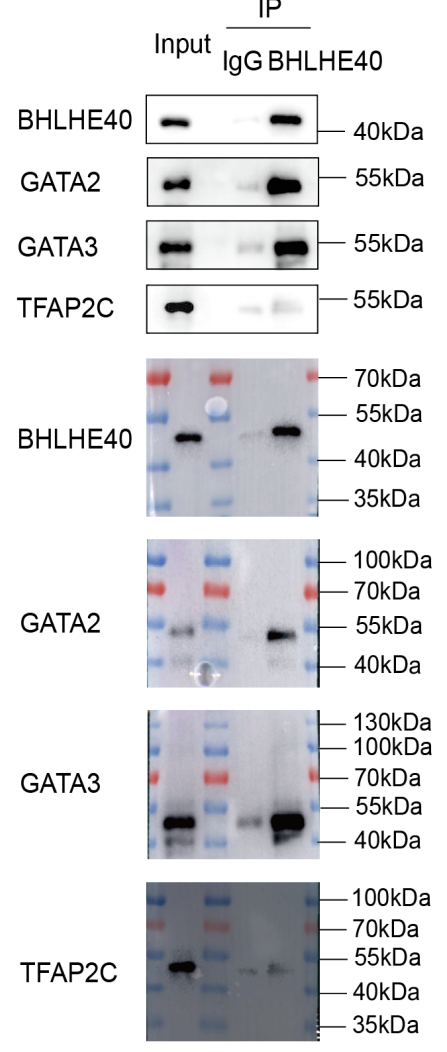

**Figure S4A**

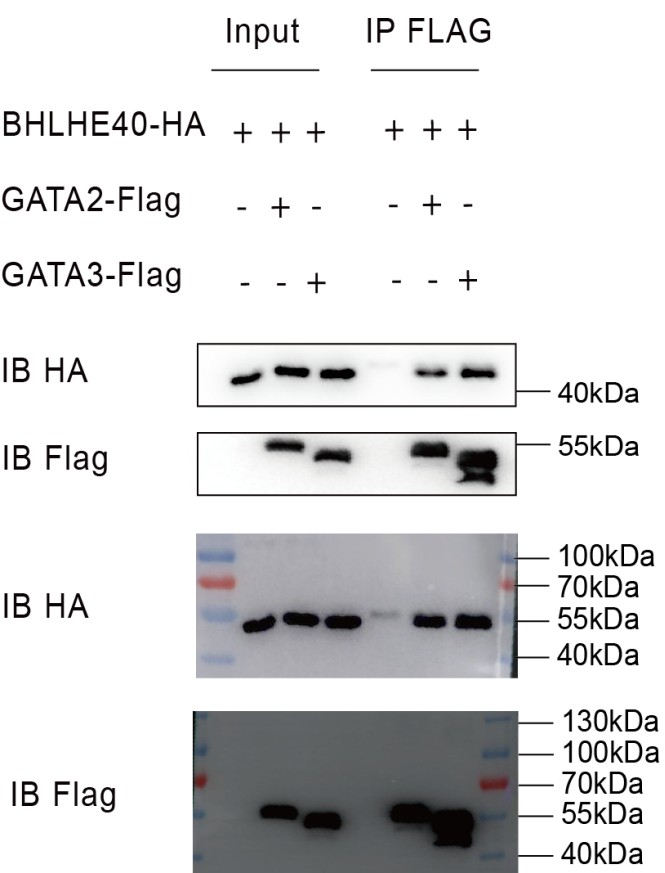

**Figure S4B**

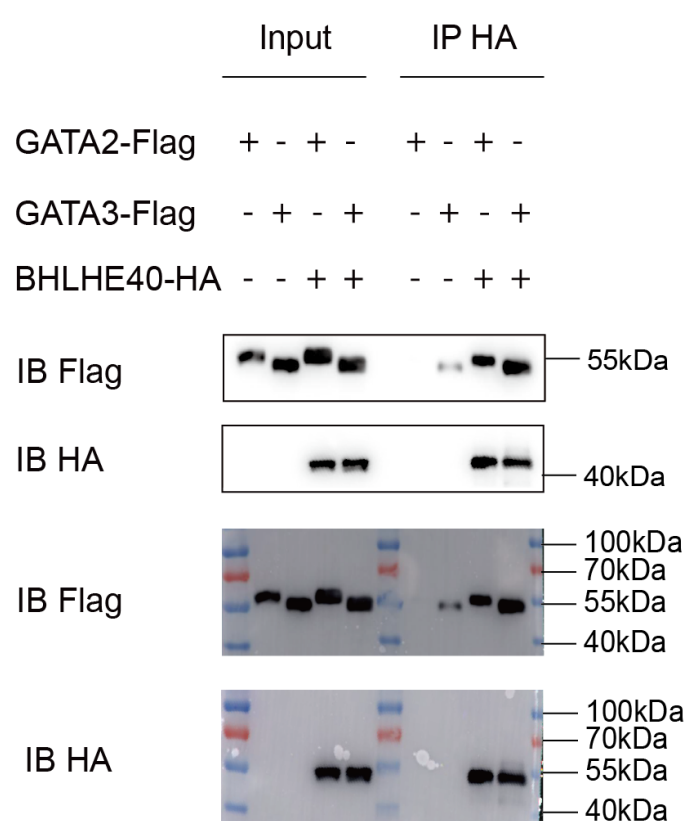

Supplement: Supplementary file 2 — Supporting Data [file ADVS-12-e07642-s002.pdf]
